# Crystal structures of *Moorella thermoacetica* cyanuric acid hydrolase reveal conformational flexibility and asymmetry important for catalysis

**DOI:** 10.1371/journal.pone.0216979

**Published:** 2019-06-10

**Authors:** Ke Shi, Seunghee Cho, Kelly G. Aukema, Thomas Lee, Asim K. Bera, Jennifer L. Seffernick, Lawrence P. Wackett, Hideki Aihara

**Affiliations:** 1 Department of Biochemistry, Molecular Biology and Biophysics, University of Minnesota, Minneapolis, Minnesota, United States of America; 2 BioTechnology Institute, University of Minnesota, St. Paul, Minnesota, United States of America; 3 Department of Biochemistry, University of Colorado Boulder, Boulder, Colorado, United States of America; 4 Microbial and Plant Genomics Institute, University of Minnesota, St. Paul, Minnesota, United States of America; University of Parma, ITALY

## Abstract

An ancient enzyme family responsible for the catabolism of the prebiotic chemical cyanuric acid (1,3,5-triazine-2,4,6-triol) was recently discovered and is undergoing proliferation in the modern world due to industrial synthesis and dissemination of 1,3,5-triazine compounds. Cyanuric acid has a highly stabilized ring system such that bacteria require a unique enzyme with a novel fold and subtle active site construction to open the ring. Each cyanuric acid hydrolase monomer consists of three isostructural domains that coordinate and activate the three-fold symmetric substrate cyanuric acid for ring opening. We have now solved a series of X-ray structures of an engineered, thermostable cyanuric acid ring-opening enzyme at 1.51 ~ 2.25 Å resolution, including various complexes with the substrate, a tight-binding inhibitor, or an analog of the reaction intermediate. These structures reveal asymmetric interactions between the enzyme and bound ligands, a metal ion binding coupled to conformational changes and substrate binding important for enzyme stability, and distinct roles of the isostructural domains of the enzyme. The multiple conformations of the enzyme observed across a series of structures and corroborating biochemical data suggest importance of the structural dynamics in facilitating the substrate entry and the ring-opening reaction, catalyzed by a conserved Ser-Lys dyad.

## Introduction

**Fig 1:**
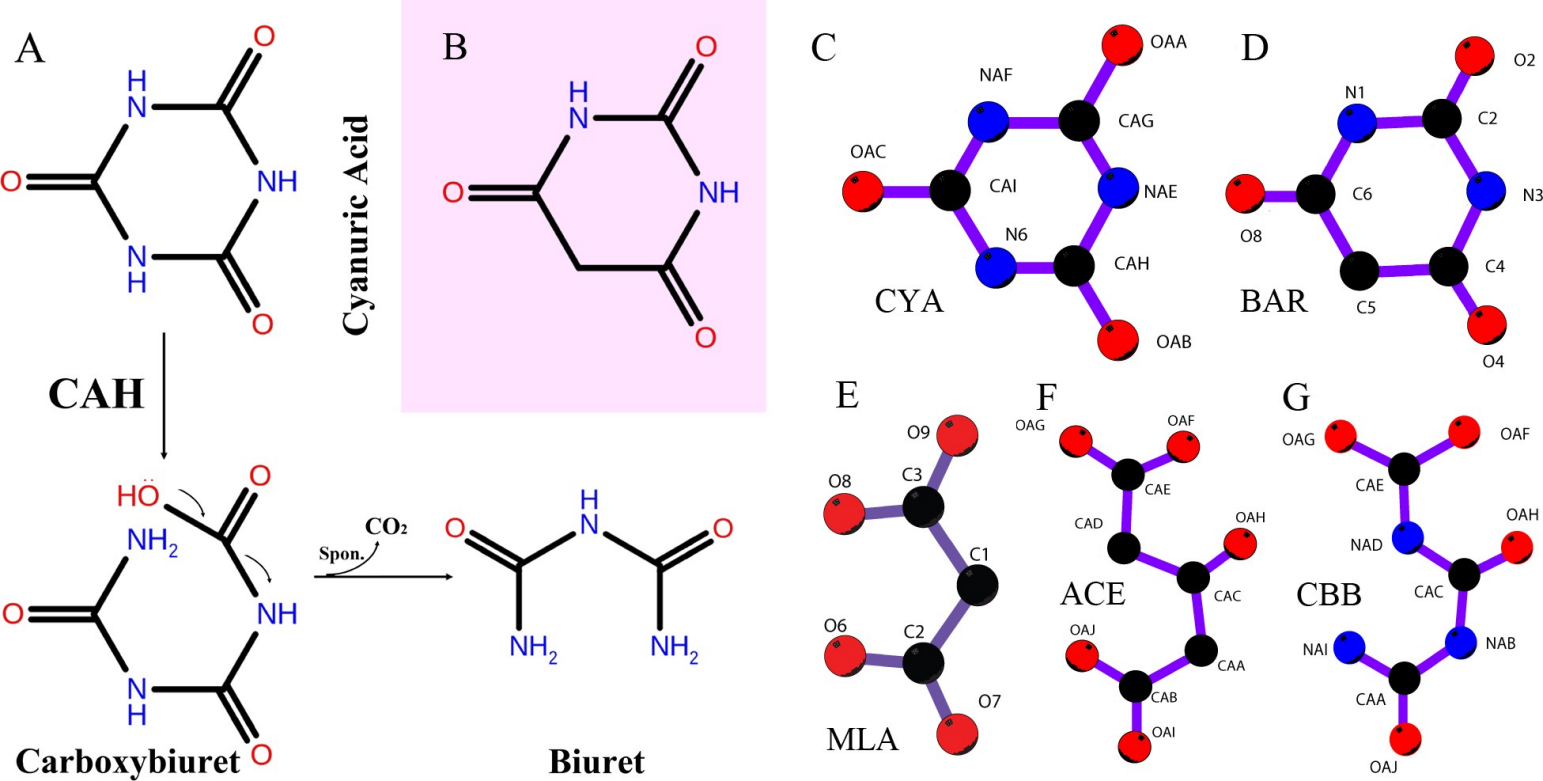
A. Schematic representation of the ring opening reaction catalyzed by Cyanuric Acid Hydrolase (CAH). B. Barbituric acid, differing from cyanuric acid by only one non-hydrogen atom, inhibits the CAH activity. C-G. The chemicals discussed in the paper, either used for soaking, or in the crystallization solution or enzymatic reaction intermediates. C. Cyanuric acid, substrate of the enzyme. D. Barbituric acid, a tight binding iso-structural inhibitor of the enzyme. E. Malonate, one of the components used in crystallization, a mimic of the final product of the enzymatic reaction, biuret. F. 3-Oxopentanedioic acid, or 1,3-Acetonedicarboxylic acid, a mimic of the open-ring intermediate, designed for soaking to form enzyme-intermediate complex. G. The theoretical open-ring intermediate generated by the enzymatic reaction.

Three hundred million pounds of cyanuric acid (1,3,5-triazine-2,4,6-triol, Fig 1A and 1C) are produced industrially due to its facile synthesis, high stability, and utility in the production of herbicides and disinfectants[1]. For disinfection, it is used directly to stabilize sodium hypochlorite or can be delivered as N,N,N-trichloroisocyanuric acid to provide both disinfection and stabilization in an aqueous environment. Nature predated anthropogenic cyanuric acid synthesis by billions of years with the compound being found in space[2] and *s*-triazines being formed in prebiotic chemistry experiments from simple precursors[3].

Due to the chemical’s ubiquitous and ancient presence on earth, the enzymes that uniquely act on it, cyanuric acid hydrolases (Fig 1A), are thought to be of ancient origin[4, 5]. The ancient origin of cyanuric acid hydrolase is supported by bioinformatics analysis, which shows sequence divergence tracking with phylogeny. The enzyme is only found in bacteria and fungi, is in a unique protein family, and is rare. Less than one percent of sequenced contemporary bacteria and fungi show evidence for cyanuric acid hydrolase gene homologues. However, a cluster of highly related sequences is found in divergent organisms that biodegrade the more than one billion pounds of industrial 1,3,5-triazine ring compounds, suggesting that the cyanuric acid hydrolase gene is undergoing a modern resurgence[6, 7].

In the last few years the first structures of a cyanuric acid hydrolase have been reported[8–10]. The two structures reported were for the enzymes from *Pseudomonas* sp. ADP, isolated for growth on the *s*-triazine herbicide atrazine, and from *Azorhizobium caulinodans*, in which the gene was discovered by genome mining. The two enzymes share 56% sequence identity and comprise a novel fold characterized by a monomer with three separated but structurally homologous domains converging to form the active site that binds and activates the three-fold symmetric substrate. The structures have shown that three Ser-Lys dyads are symmetrically arranged in the active site. Based on the structural data, the ring opening enzyme was proposed to catalyze a nucleophilic attack by a Ser γO which is deprotonated by the nearby Lys, producing a serine-ester covalent enzyme intermediate. One of the Ser-Lys dyads was proposed to preferentially initiate the reaction, although conceptually any of them could potentially catalyze the reaction. Three Arg residues in the active site aid the reaction by orientating the substrate and stabilizing the intermediates, the acyl-enzyme and the carboxybiuret produced by hydrolysis of acyl-enzyme. Subsequent decarboxylation of the enzymatic product has been proposed to be spontaneous based on detecting substoichiometric carboxybiuret off the enzyme surface, but the potential for enzyme assistance in decarboxylation is not precluded[6]. However, owing to the limited number and types of ligands (all planar with either strict (Fig 1A and 1C) or pseudo 3-fold (Fig 1B and 1D) [8, 9], or non-planar but still with pseudo 3-fold[10] in homologous barbituric acid hydrolase) used in the obtained complex structures, as well as moderate resolution in most cases (S1 Table), our knowledge on the active site and the catalytic mechanism of these enzymes is still limited.

Cyanuric acid degradation has become an industrial focus due also to its use in stabilizing chlorine disinfectant in pools and spas to prevent the spread of water-borne diseases[11, 12]. Chlorine dissipates over time but cyanuric acid gets accumulated, due to its extreme chemical stability and non-volatility. This necessitates drainage and replacement with fresh water, posing a disposal issue and a significant loss of potable water in hot-dry environments. The use of cyanuric acid hydrolase to solve this societal problem is being explored and the best candidate for this biotechnological application is the thermostable enzyme from *Moorella thermoacetica*[13, 14].

In this context, the X-ray structure of the *Moorella* cyanuric acid hydrolase was highly sought to further study the catalytic mechanism of this enzyme family and to understand and design parameters for commercial applications. Here, we are reporting a series of structures of *Moorella* cyanuric acid hydrolase, including complexes with various bound ligands: substrate, inhibitor, and reaction intermediate analogue. These structures rationalize the tetrameric architecture, elucidate the metal requirement, and highlight asymmetry in the pseudo-symmetrical active site. Furthermore, the open *vs*. closed conformation and subtle structural differences between individual monomers within the tetramer upon binding of various ligands shed light on the dynamical properties of the enzyme that are potentially important in substrate binding and catalysis.

## Materials and methods

### Designing morella CAH for crystallization

The surface entropy reduction method was applied to crystallize *Morella* CAH, using both the XtalPred[15] and SERp[16] servers. Residues 279–292 were predicted to be disordered. This disordered loop region “LKFDCCPPAEELAK” in MCAH was replaced with the loop region “IRSDDEMDR” from AtzD, which formed a small helix in the AtzD crystal structure (PBD Code 4BVQ). Additionally, Q103, E104, and K107 were mutated to alanines to reduce conformational entropy. The redesigned *Moorella* cyanuric acid hydrolase (RMCAH) is 5 amino acids shorter than the wild-type MCAH. The DNA sequence corresponding to the designed amino acid sequence was synthesized and cloned in pET28b+ by GenScript (Piscataway, NJ).

### Protein expression and purification

The wild-type *Morella* CAH was purified as described previously [6]. The engineered full-length *Morella* CAH (RMCAH) gene was expressed in *Escherichia coli* BL21 (DE3) from the pET28b+ based construct. Cells were grown at 37°C in LB medium (10 g Bacto tryptone, 5 g yeast extract, 10 g NaCl per liter) supplemented with 50 μg/ml kanamycin and induced with 0.5 mM of isopropyl β-D-1-thiogalactopyranoside (IPTG) at a final concentration for 30 hours at 20°C.

After collection by centrifugation (6400 × g, 10 min, 4°C), the cells were resuspended in binding buffer (50 mM Tris–HCl pH 7.0, 200 mM NaCl, 5 mM imidazole). Sonication was used for cell disruption and the cell lysate was centrifuged at 63,988 × g for 90 min at 4°C. After centrifugation, the cell lysate was applied to a Ni–NTA resin (Thermo scientific) packed in a column that had been equilibrated with the binding buffer. After removing unbound protein with washing buffer (50 mM Tris–HCl pH 7.0, 200 mM NaCl, 10 mM imidazole), RMCAH was eluted with four column volumes of elution buffer (50 mM Tris–HCl pH 7.0, 200 mM NaCl, 200 mM imidazole). For further purification, gel filtration chromatography was performed on a Biologic Duoflow system (Bio-RAD) using a Superdex 200 10/300 GL gel-filtration column (GE Healthcare) equilibrated with a running buffer consisting of 20 mM Tris–HCl pH 7.0, 200 mM NaCl. The enzyme was concentrated by ultrafiltration (EMD, Amicon) and the concentration of the enzyme was determined with a NanoDrop 8000 UV–Vis spectrophotometer (Thermo Scientific). Expression plasmids for the RMCAH mutants (E301A, G53C/E235C, and C46S/C162A/C218V/G53C/E235C) were generated by the standard site-directed mutagenesis procedure and the mutated proteins were expressed and purified as described above for RMCAH.

### Enzyme activity assays

Activities and kinetic constants of the WT *Moorella* CAH and re-engineered *Moorella* CAH (RMCAH) in 25 mM Tris pH 8.0 were determined according to the methods detailed in Seffernick et al. 2012 [6]. For testing protein thermal stability, the activity of 26 μM RMCAH and its E301A variant in phosphate buffered saline at pH 7.2 were determined after incubation at 62°C for 30 minutes. The ring cleaving cyanuric acid hydrolysis reaction was assayed by measuring the decrease in absorbance of cyanuric acid at 214 nm. The activities of RMCAH and RMCAH-CC (G53C/E235C) in the presence or absence of 10 mM dithiothreitol (DTT) were compared for cyanuric acid removal using a modified melamine precipitation assay [17] Briefly, 0.3 μM RMCAH and RMCAH-CC enzymes were incubated with 140 ppm cyanuric acid in phosphate buffered saline at pH 8.0 (137mM NaCl, 2.7mM KCl, 10mM Na_2_HPO_4_, 1.8mM KH_2_PO_4_). At each time point, a fraction of the reaction mixture was removed and mixed thoroughly with an equal volume of melamine solution (2.5 mg/mL in water). The cyanuric acid concentration was determined by measuring turbidity at 600 nm and comparing it to the standard curve to quantitate melamine cyanurate precipitation.

### Thermal melting monitored by circular dichroism (CD) spectroscopy

For far-UV CD data collection, the measurements were performed on a Jasco J-815 spectrometer with 25 μM protein in 50 mM Tris-HCl buffer, pH 7.2 in a 1 mm pathlength cell. For thermal melting, the changes in molar ellipticity at 220 nm of the proteins were sampled at 1°C increment during continuous heating of the solutions from 55 to 90°C with a ramp rate of 1°C per minute.

### Crystallization and crystals handling

The enzyme was concentrated to 12 mg/ml. The initial crystallization screening of RMCAH with barbituric acid or cyanuric acid was carried out on a Rigaku CrystalMation system at the Nanoliter Crystallization Facility of the University of Minnesota using the sitting-drop vapor-diffusion method. Crystals appeared in the condition containing 8% tacsimate (Hampton Research) and 20% PEG3350 in 2 weeks and were harvested after 3 weeks. The crystals grown without divalent metal ion diffracted to ~3.2 Å resolution. An additive screening identified a crystal form with altered growth behavior in the presence of 100 mM CaCl_2_. Subsequent manually set hanging drop plates produced crystals suitable for diffraction data collection. The crystals took more than 2 months to appear in the CaCl_2_ containing condition. To facilitate the process, seed crystals were generated and introduced to the drops 4 days after the crystallization drops were set up. Crystals could appear within another week. Owing to the tight binding of MLA to the active site of the enzyme, the APO structure was obtained by a series of soaking with reduced MLA concentration until no MLA is presented in the soaking buffer. The obtained APO crystals were used to soak with other ligands. To crystallize RMCAH (C46S/C162A/C218V/G53C/E235C) mutant, ~20 mg/ml protein was mixed in 1:1 volume ratio with a well solution consisting of 0.2M tri-ammonium citrate and 20% w/v PEG3350. Crystals appeared in 6 days. The crystals were extremely fragile and diffracted at best to only 3.3 Å resolution. The crystal contained 8 protein monomers in the asymmetric unit.

### Data collection and structural solution

The X-ray diffraction data from flash-cooled crystals were collected at beamlines 24-ID-C and 24-ID-E of the Advanced Photon Source (APS). Data were processed using HKL2000 or XDS[18, 19]. Crystals belonged to the P2_1_2_1_2_1_ space group and diffracted to a resolution limit ranging from 1.51 Å to 2.25Å. Molecular replacement was performed with Phaser[20], using a monomer of the previously determined ACAH structure[9] (PDB Code 4NQ3) as the search model. Four copies (or eight copies for SAVCC structure) of the protein in the asymmetric unit were sequentially located. The coordinates for malonate were obtained from protein data bank and restrains generated by PHENIX[21]. Iterative cycles of model building and refinement were conducted using COOT[22] and PHENIX refinement modules. Refinement of the model against X-ray data was carried out until convergence was achieved. Active site configurations were confirmed by simulated-annealing omit map. A summary of the data collection and model refinement statistics is shown in Table 1. MolProbity[23, 24], and PROCHECK[25] were used for structure quality analysis. Structure figures were generated with PyMOL (http://www.pymol.org), CCP4MG[26] and UCSF CHIMERA[27]. The atomic coordinates and structure factors have been deposited in the Protein Data Bank (PDB) with the accession codes 6BUM (malonate: MLA), 6BUN (APO), 6BUO (reconstructed MLA), 6BUP (substrate: CYA), 6CWJ (intermediate analogue: ACE), 6BUQ (BAR-a), 6BUR (BAR-b), and 6DHJ (SAVCC). The accession codes are also listed in Table 1.

**Table 1:** X-ray data collection and model refinement statistics.

	MLA	APO	MLA-R	CYA	ACE	BAR-a	BAR-b	SAVCC
**Data collection**								
Resolution range	30.0–1.51 (1.56–1.51)	75.2–1.78 (1.84–1.78)	29.9–1.85 (1.92–1.85)	81.5–1.88 (1.90–1.88)	95.3–2.25 (2.33–2.25)	39.7–1.88 (1.95–1.88)	39.1–2.15 (2.23–2.15)	91.8–3.2 (3.3–3.2)
Space group	P2_1_2_1_2_1_	P2_1_2_1_2_1_	P2_1_2_1_2_1_	P2_1_2_1_2_1_	P2_1_2_1_2_1_	P2_1_2_1_2_1_	P2_1_2_1_2_1_	P2_1_2_1_2_1_
Unit cell (Å)	81.44 88.69 204.28	81.23 89.33 199.42	81.45 88.82 202.85	81.07 89.42 198.55	79.14 88.84 190.51	81.05 89.77 198.71	80.98 89.17 199.05	102.91 164.56 202.87
Protomer/ASU	4	4	4	4	4	4	4	8
Total reflections	871695 (51699)	988066 (96968)	847089 (84972)	786419 (78576)	404053 (37264)	516283 (51236)	343778 (35643)	269581 (27991)
Unique reflections	221630 (12043)	139309 (13688)	125885 (12384)	117806 (11655)	64090 (6207)	117800 (11596)	78456 (7810)	56567 (4674)
Multiplicity	3.9 (2.0)	7.1 (7.1)	6.7 (6.8)	6.7 (6.7)	6.3 (5.8)	4.4 (4.4)	4.4 (4.6)	4.8(4.9)
Completeness	0.96 (0.70)	1.00 (1.00)	1.00 (1.00)	1.00 (1.00)	0.99 (1.00)	0.99 (0.99)	0.99 (1.00)	0.99 (1.00)
*I/σ(I)*	20.1 (1.2)	17.4 (3.0)	17.6 (2.0)	13.9 (1.7)	10.6 (1.5)	11.1 (1.6)	10.6 (2.4)	10.3 (0.7)
R-merge	0.037 (0.625)	0.088 (0.705)	0.124 (1.181)	0.089 (1.13)	0.156 (1.22)	0.085 (0.976)	0.105 (0.60)	0.109 (1.84)
CC_1/2_	0.999 (0.471)	0.998 (0.808)	0.996 (0.566)	0.999 (0.586)	0.994 (0.525)	0.998 (0.595)	0.998 (0.595)	0.999 (0.39)
**Refinement**								
R-work (%)	12.50 (26.02)	14.77 (23.30)	14.94 (36.46)	14.79 (25.76)	18.60 (27.34)	15.28 (27.62)	16.19 (25.50)	20.8 (37.7)
R-free (%)	14.02 (27.29)	17.36 (26.25)	17.47 (37.72)	18.06 (30.61)	22.36 (29.84)	18.17 (31.41)	21.20 (30.42)	25.0 (40.9)
Non-hydrogen atoms	12173	12248	11934	11732	11453	11665	11255	21566
Macromolecules	10850	10821	10821	10752	10799	10775	10795	21462
Ligands	153	115	152	133	62	93	48	104
Solvent	1170	1312	961	847	592	797	412	
Protein residues	1450	1449	1448	1449	1452	1447	1449	2897
RMSD from ideal								
Bond length (Å)	0.005	0.008	0.005	0.010	0.005	0.008	0.013	0.005
Bond angle (°)	1.17	1.18	0.68	1.31	1.03	1.15	1.37	0.93
Ramachandran plot								
Favored (%)	97.6	97.6	97.3	96.7	96.1	97.1	96.8	96.7
Allowed (%)	2.4	2.4	2.7	3.3	3.9	2.9	3.2	3.3
Outliers (%)	0	0	0	0	0	0	0	0
Ave. B-factor (Å^2^)	27.84	27.98	36.22	41.22	43.29	41.33	54.53	140.1
Macromolecules	26.70	27.09	36.02	41.06	43.46	41.37	54.90	140.2
Ligands	44.08	40.18	41.61	54.91	46.59	45.28	39.60	112.4
Solvent	36.22	34.23	37.64	41.10	39.86	40.30	46.70	
**PDB ID**	6BUM	6BUN	6BUO	6BUP	6CWJ	6BUQ	6BUR	6DHJ

Statistics for the highest-resolution shell are shown in parentheses.

### Hydrogen-deuterium exchange mass spectrometry (HDX-MS)

Purified RMCAH was mixed with BAR (final 20 μM RMCAH monomer + 100 μM BAR) in 20 mM HEPES-NaOH (pH 7.4), 100 mM NaCl, 0.5 mM Tris(2-carboxyethyl)phosphine hydrochloride, and 0.2% DMSO, then diluted 10-fold with D_2_O at 10°C (final 100 μL). Aliquots were taken at 8 different time points, 0.5, 1, 3, 5, 10, 30, 60 and 120 minutes. Samples were quenched by addition of an equal volume of an ice-cold quench buffer (1.25% formic acid, 1.5 M guanidine HCl), and analyzed on a Waters Synapt G2 mass spectrometry coupled to a Waters ACQUITY HDX LC system. The LC system was maintained at 0°C except for a separated chamber for the pepsin digestion (10°C). The samples were digested on a Prozyme pepsin cartridge (2.1 mm x 3.0 mm, Applied Biosystems), trapped, and desalted on a Waters ACQUITY BEH C18 VanGuard Pre-column (130Å, 1.7 μm, 2.1 mm x 5 mm) for 3 min with a flow rate of 100 μL/min Buffer A (0.1% formic acid in water). The resulting peptic peptides were resolved on a Waters ACQUITY UPLC BEH C18 column (1.7 μm, 1.0 x 100 mm) with a linear gradient ranging from 5 to 60% acetonitrile + 0.1% formic acid in 6 min at 40 μL/min. Deuteron incorporation to each peptic peptide was calculated using Waters DynamX software (version 3.0.0). The control sample did not contain BAR but had the same DMSO content. BAR up to 0.5 mM did not cause measurable change in the pH of this buffer. To qualitatively present the effect of BAR binding on the HDX rate for various regions in the RMCAH structure, the data from 0.5 and 60 minutes were used to calculate the HDX rate difference, which was assigned as the b-factor for the corresponding peptide segment.

## Results

### Overall structure

The wild-type *Moorella thermoacetica* Cyanuric Acid Hydrolase (MCAH) failed to crystallize despite extensive screening. The enzyme was therefore subsequently re-engineered (Re-engineered MCAH: referred to as RMCAH hereinafter) by applying surface entropy reduction and loop reconstruction methods, producing a fully active enzyme five amino acids shorter than the wild-type MCAH. RMCAH and wild-type MCAH showed indistinguishable specific activities and enzyme kinetics within error (S1 Fig). The RMCAH was readily crystallized. However, the crystals initially obtained only diffracted to ~3.2 Å, with six tetramers (twenty-four monomers) in the asymmetric unit. Subsequent screening identified Ca^2+^ as a chemical additive that can improve crystal quality. With 100 mM CaCl_2_ present in the crystallization solution, the diffraction of the crystals drastically improved and the volume of the asymmetric unit was reduced to contain only one tetramer.

**Fig 2:**
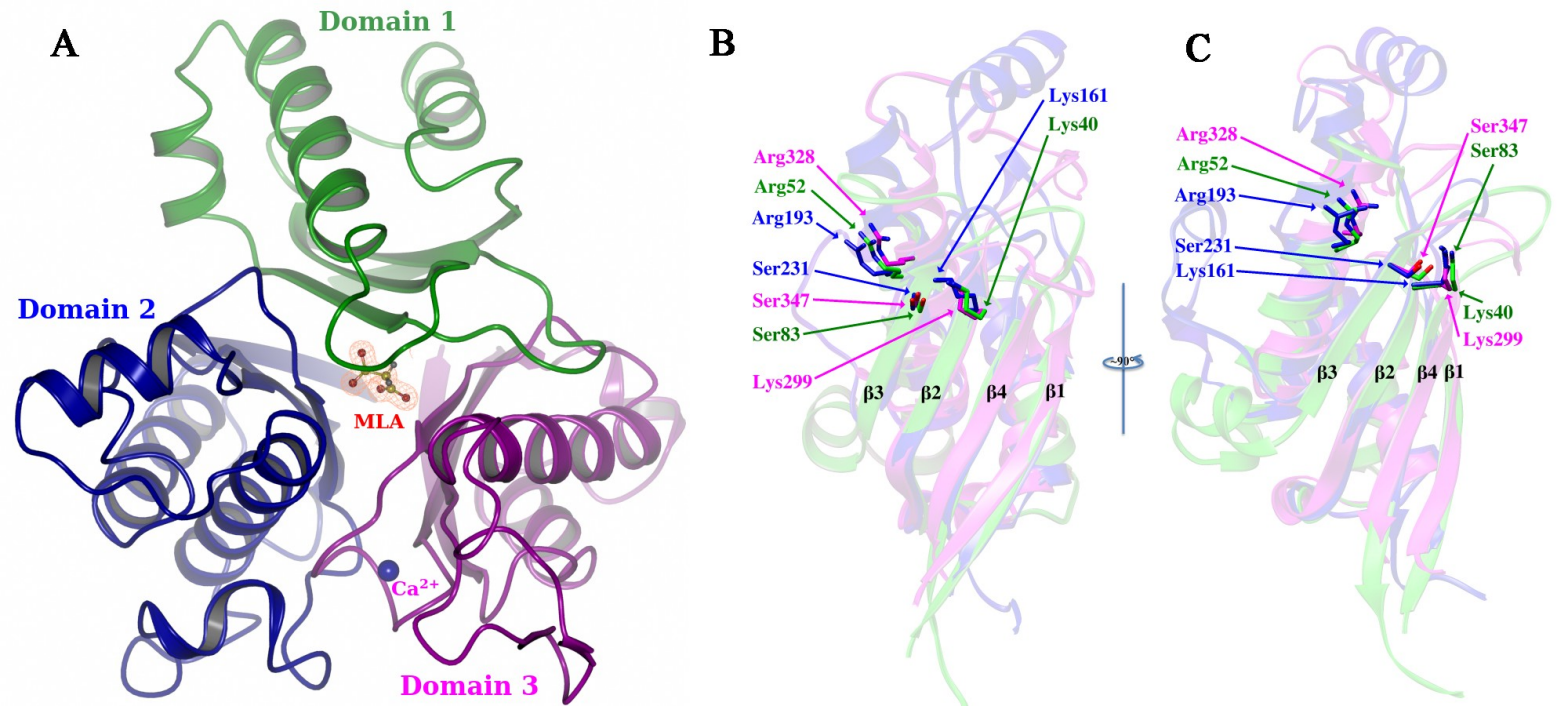
Domain architecture of cyanuric acid hydrolase. A. RMCAH monomer shown in a ribbon diagram viewed along the pseudo three-fold axis and with the three domains colored differently: Domain 1 (residues 1–102) is in green, Domain 2 (111–247) in blue and Domain 3 (253–367) in magenta. Malonate observed at the domain interface is shown in a ball-and-stick representation. The 2mFo-DFc electron density map contoured at 1.0 σ level is overlaid and shown in orange-colored mesh. B. A superposition of the three domains color-coded as in A with the conserved Ser-Lys dyads and the partner Arg side chains shown in sticks. The arginine is located in the middle of the longest helix of each domain, while the serine and lysine are from the 3^rd^ and 2^nd^ β-strands of the 4-β-pleated core, respectively. The three corresponding serines superpose very well, so do the three lysines and arginines. C. Same as B with a view rotated ~90° about the vertical axis.

The X-ray crystal structure of the engineered *Moorella* cyanuric acid hydrolase was determined by molecular replacement and refined to 1.51 Å resolution. The structure shows that each RMCAH monomer consists of a β-barrel-like core surrounded by α-helices (Fig 2A, S2 Fig), resembling the *Azorhizobium caulinodans* ORS 571 Cyanuric Acid Hydrolase[9] (ACAH, PDBID 4NQ3) and *Pseudomonas* sp. ADP AtzD[8] structures (PDBID 4BVQ) with a backbone r.m.s.d. of 1.2 Å and 1.0 Å, respectively. Consistent with the “Toblerone fold” observed for these enzymes[8–10], the RMCAH monomer is composed of three concatenated, highly divergent, but structurally homologous domains, which endows the enzyme monomer an internal pseudo-three-fold symmetry (Domains 1, 2, 3 shown in Fig 2A). Protein sequence identities and r.m.s.d. of the backbone atoms between the three domains are: domains 1 and 2 having 11% and 3.7 Å, domains 1 and 3 having 8% and 2.7 Å, and domains 2 and 3 having 21% and 1.9 Å. The domains 1 and 3 are similar in size (102 and 110 residues) whereas domain 2 is slightly larger (137 residues). The residues previously shown to be critical for catalysis in the CAH homologs, including the Ser-Lys dyad and an accompanying Arg residue from each domain, show similar spatial positions upon superposition of the three domains (Fig 2B and 2C).

### Tetramer architecture

**Fig 3:**
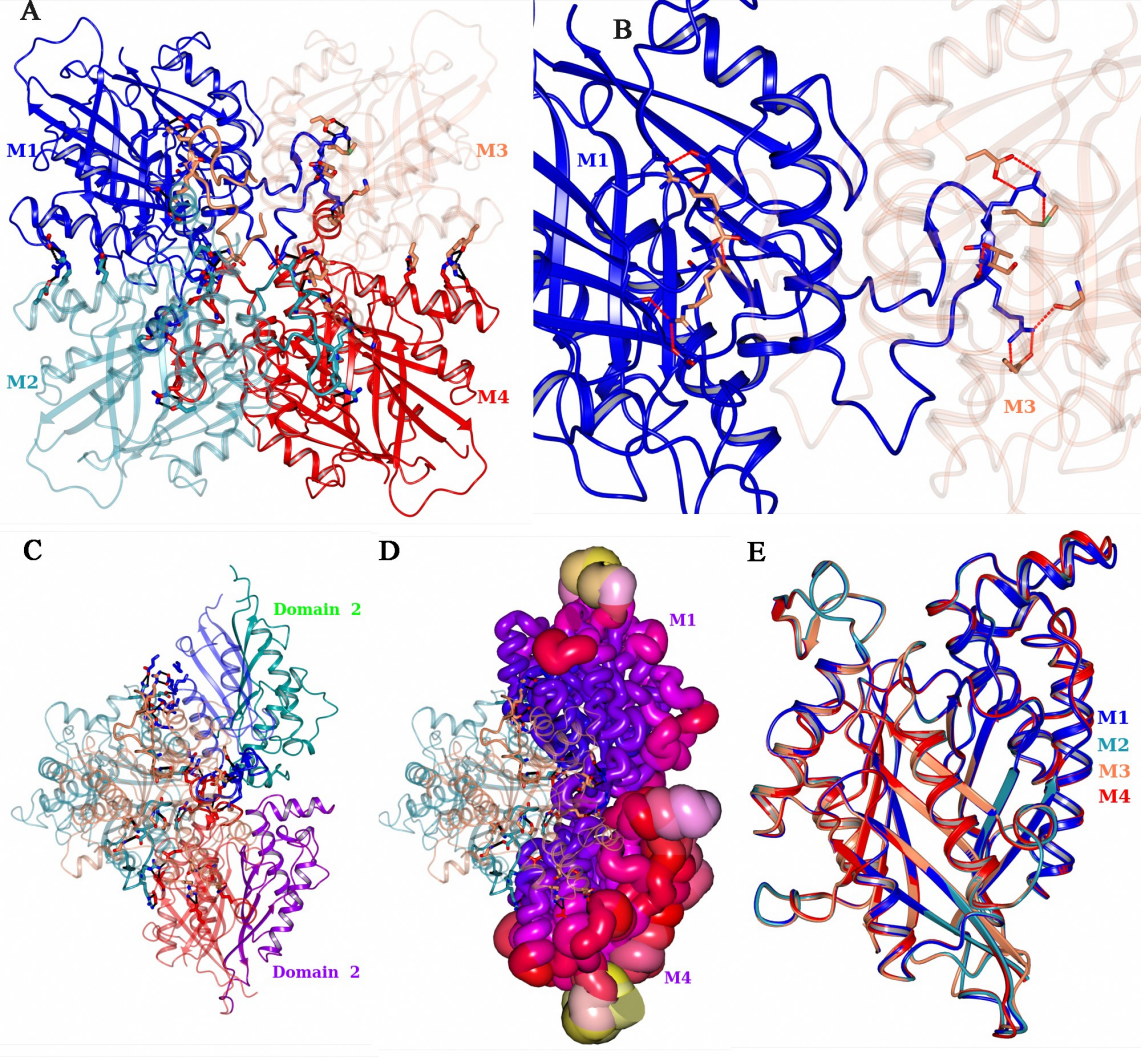
Architecture and properties of the RMCAH tetramer. A. Domain 3 and domain 1 form the tetramer core. An extended loop from the domain 3 forms extensive inter-monomer interactions. B. The detailed polar interactions made by the extended loop of domain 3 in the tetramer core. C. Domains 2 of monomer M4 depicted in purple and that of M2 depicted in green, showing their lack of inter-monomer interactions in the tetramer. D. M2 and M4 are shown in B-factor scaled worm. The color with increased B-factor is depicted in blue<red<pink<yellow. E. A superposition of the four monomers from the parental RMCAH/MLA structure. The four monomers have very similar conformation, only M1 has small difference at the α-helix formed by residues 167–176 (upper right corner).

RMCAH forms a stable tetramer in solution, based on size exclusion chromatography and dynamic light scattering analysis (data not shown). The analysis of our crystal structure by PISA[28] predicts an average free energy of -26.9 kcal/mol for the tetramer formation. The buried surface area (BSA) and solvent accessible surface area (ASA) for a tetramer are 19288 and 43881 Å^2^, respectively. The extensive contact supports the RMCAH biological unit being a tetramer form. The tetramer has a 222 internal point symmetry as observed previously for AtzD[8] and ACAH[9]. Interestingly, molecular interfaces within the tetramer are mostly formed by domain 1 and domain 3 (Fig 3A and 3B), whereas domain 2 is least involved in tetramer association (Fig 3C) and shows relatively higher B-factors (Fig 3D, S2 Table). There is a total of 225 amino acids located at the molecular interface, to which domain 2 only contributes 13 non-aromatic residues, namely Ala, Thr, Asp, Glu and Lys residues which are mainly engaged in polar contacts. The four monomers have very similar conformations, only showing small differences in the positioning of the α-helix formed by residues 167–176 (Fig 3E).

### The enzyme active site

**Fig 4:**
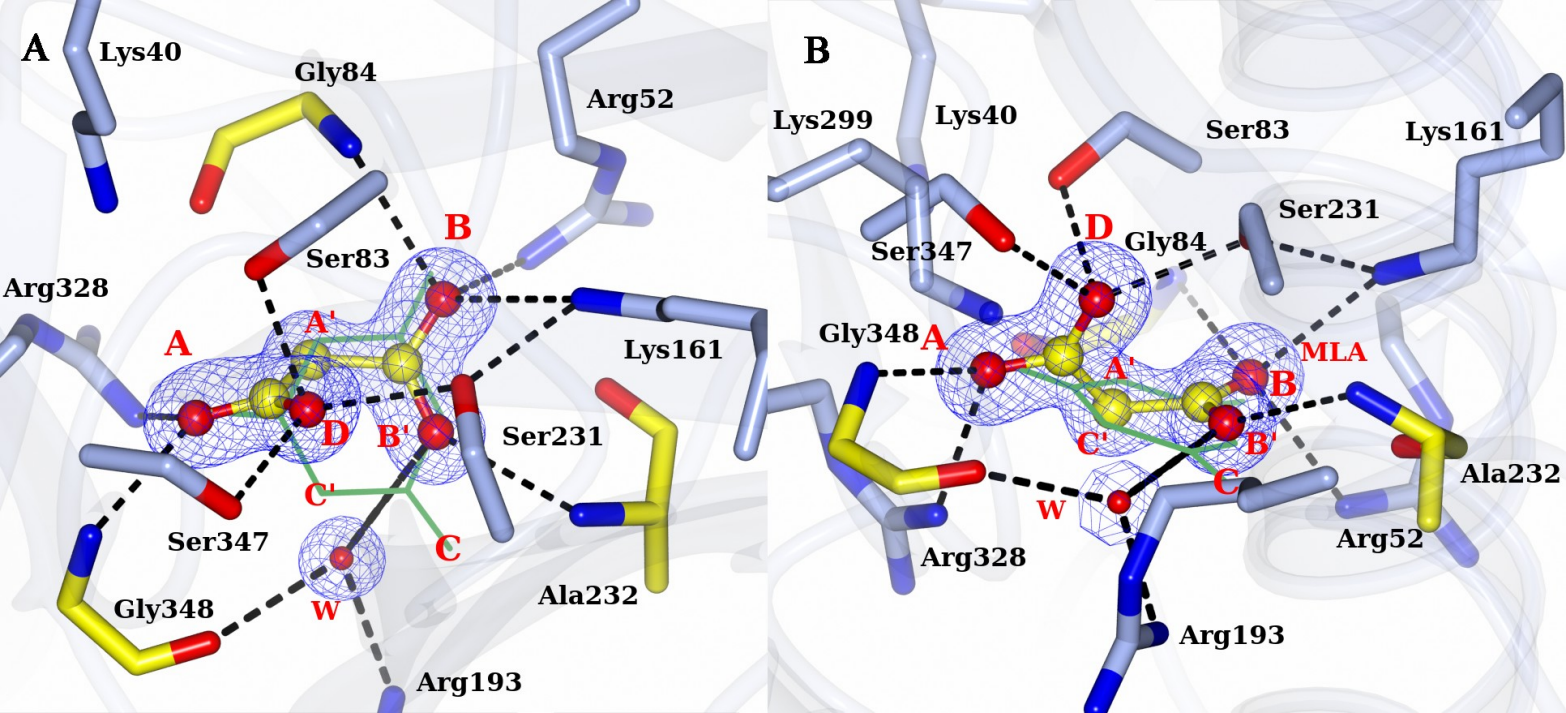
Positioning of MLA in the RMCAH active site. A. Top-view of MLA in the active site overlaid with 2mFo-DFc electron density map contoured at 1.0 σ level, depicted in blue mesh. A barbituric acid (BAR) molecule from RMCAH/BAR structure (depicted in green lines, will be discussed in detail later) is depicted as a reference to show the substrate plane and demonstrate the relative location of MLA atoms. The three carbonyl oxygen positions of BAR are designated A, B, and C for simplifying structural descriptions. The three intervening atoms corresponding to amide nitrogen atoms in CYA are named A’, B’, and C’. B. Side-view of interactions between malonate and RMCAH active site residues.

As observed for AtzD[8] and ACAH[9], the active site of RMCAH lies on the pseudo-three-fold axis within each monomer and is formed by similar sets of residues from all three domains. To facilitate description of the RMCAH structures with various ligands in the active site, we will define nomenclatures for positions within the active site. An Arg residue and a neighboring backbone amide group from each domain form a positively charged binding pocket. These sites from domain 3, 1, and 2 are designated A, B, and C, respectively (Fig 4A and 4B). As described later in RESULTS and based on the previously reported AtzD[8] and ACAH[9] structures, these oxyanion holes A, B, and C accommodate the carbonyl oxygen atoms of the substrate cyanuric acid (CYA) (Fig 1C) or its analog barbiturate (BAR) (Fig 1B and 1D). In addition, three distinct binding sites are formed between A, B, and C and designated A’, B’, and C’. These (A’, B’, and C’) sites accommodate the amide nitrogen atoms of CYA (α-carbon atom for BAR). The three active site Ser (83, 231 and 347) γO atoms are ~ 3.9 Å from each other forming a near equilateral triangle, which parallels, but is significantly (~1.5 Å) offset from that formed by connecting the A-B-C or A’-B’-C’ positions.

### Malonate (MLA) bound structure

All four monomers within the RMCAH tetramer showed clear electron density for a bound ligand in the active site, regardless of whether or not substrate/inhibitor was added during crystallization. The ligand was readily interpreted as malonate (Fig 1E), which is a major component of tascimate used in the crystallization condition, and no residual mFo-DFc density was left after further refinement. Fig 4 shows detailed interactions formed between MLA and RMCAH active site residues. All four MLA molecules adopt a staggered conformation, in contrast to the planar/eclipsed conformation seen in AtzF[29]. The top-view of MLA (Fig 4A) shows that the MLA-protein interactions mimic the substrate analogue barbiturate (BAR)-protein interactions at A and B carbonyl-binding sites. The oxygen atom at site A almost has the same position as the substrate oxygen atom. At position B’, a carboxyl oxygen atom is hydrogen bonded with the Ala232 main chain oxygen atom. The side-view of MLA in Fig 4B shows that the two oxygen atoms of MLA, at sites B and B’, are ~ 1 Å above the substrate plane. The forth oxygen atom is far out of the plane, occupying the position D, (position D, Fig 4A and 4B) and roughly points to the center of the triangle formed by Ser (83, 231 and 347) γO atoms and is within hydrogen-bonding distance from all three Ser γO atoms. The triangle formed by the three Ser (83, 231 and 347) γO atoms is slightly expanded compared to that in structures with the position D unoccupied (S3 Table). The position D was previously reported as being occupied by a phosphate group oxygen in the AtzD[8] structure (4BVQ). A water molecule is located near the position C’ (1.3 Å away and 0.6 Å below the substrate plane, interacting with one carboxylate group oxygen atom at position B’) in the MLA-bound active site. The water molecule also forms hydrogen bonds with Arg193 NH1 and Gly348 carbonyl oxygen atoms.

### RMCAH “APO” structure and open conformation

**Fig 5:**
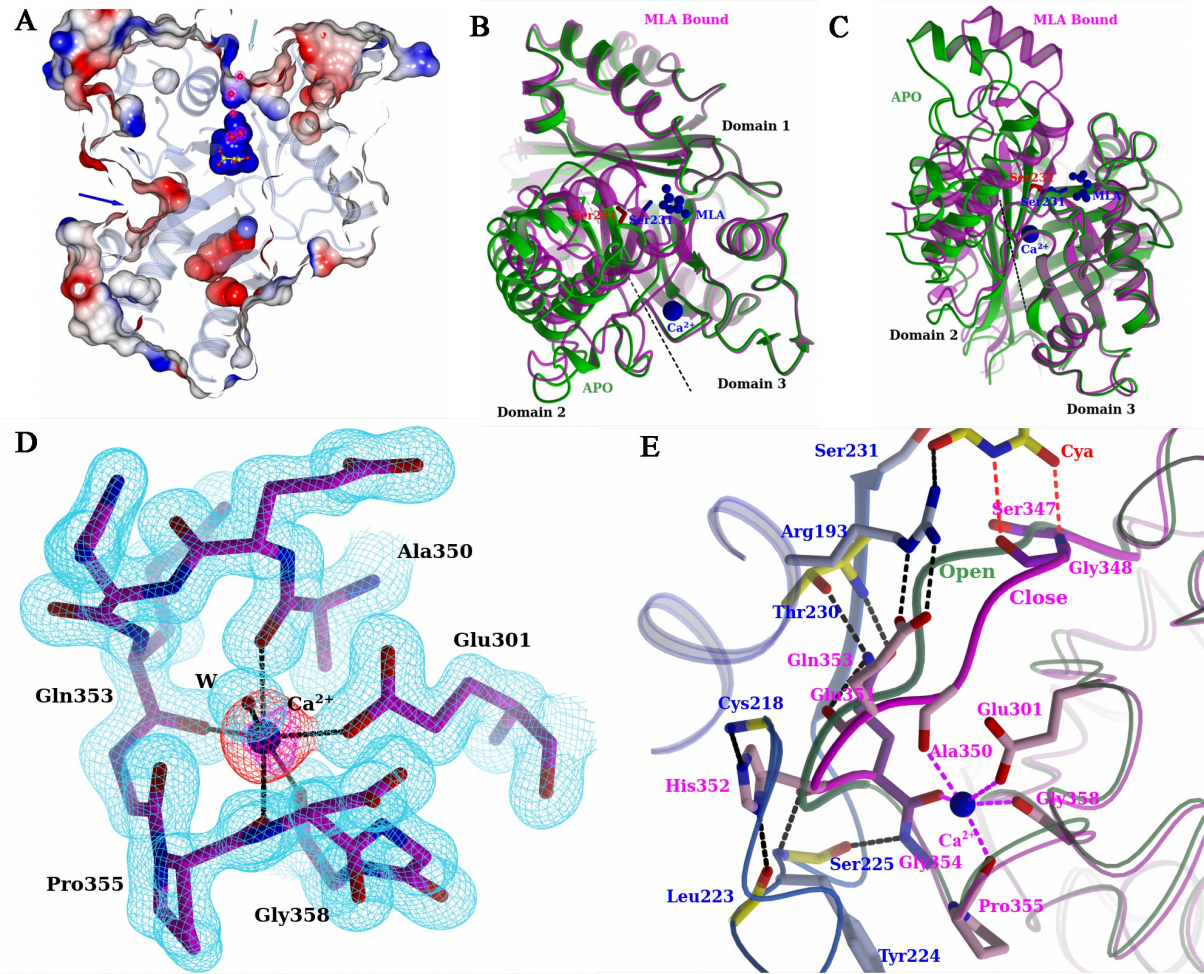
Comparison of the open and closed conformations of RMCAH, and the relationship between Ca^2+^ ion binding and RMCAH conformation. A. Molecular surface of the RMCAH/MLA complex, colored according to electrostatic potential. Two channels differ greatly in their charge properties. B. Top view of superposition of the APO open conformation (green) with the MLA-bound closed conformation (magenta). C. Side view of the superposition, with the domain 3 in front and facing to the right, showing displacement of the domain 2. See also S1 Video. D. 2mFo-DFc simulated annealing composite omit map showing the calcium binding loop contoured at 1.5σ level. The same map for Ca^2+^ is shown in orange color. The anomalous difference Fourier map for Ca^2+^ is shown in pink and contoured at 3σ level. E. A close-up view of the interactions made by the short segment Ser347-Gly348-Gly349-Ala350-Glu351-His352-Gln353 in domain 3 in the closed conformation (magenta). The backbone trace for the open conformation is shown in green for comparison.

**Fig 6:**
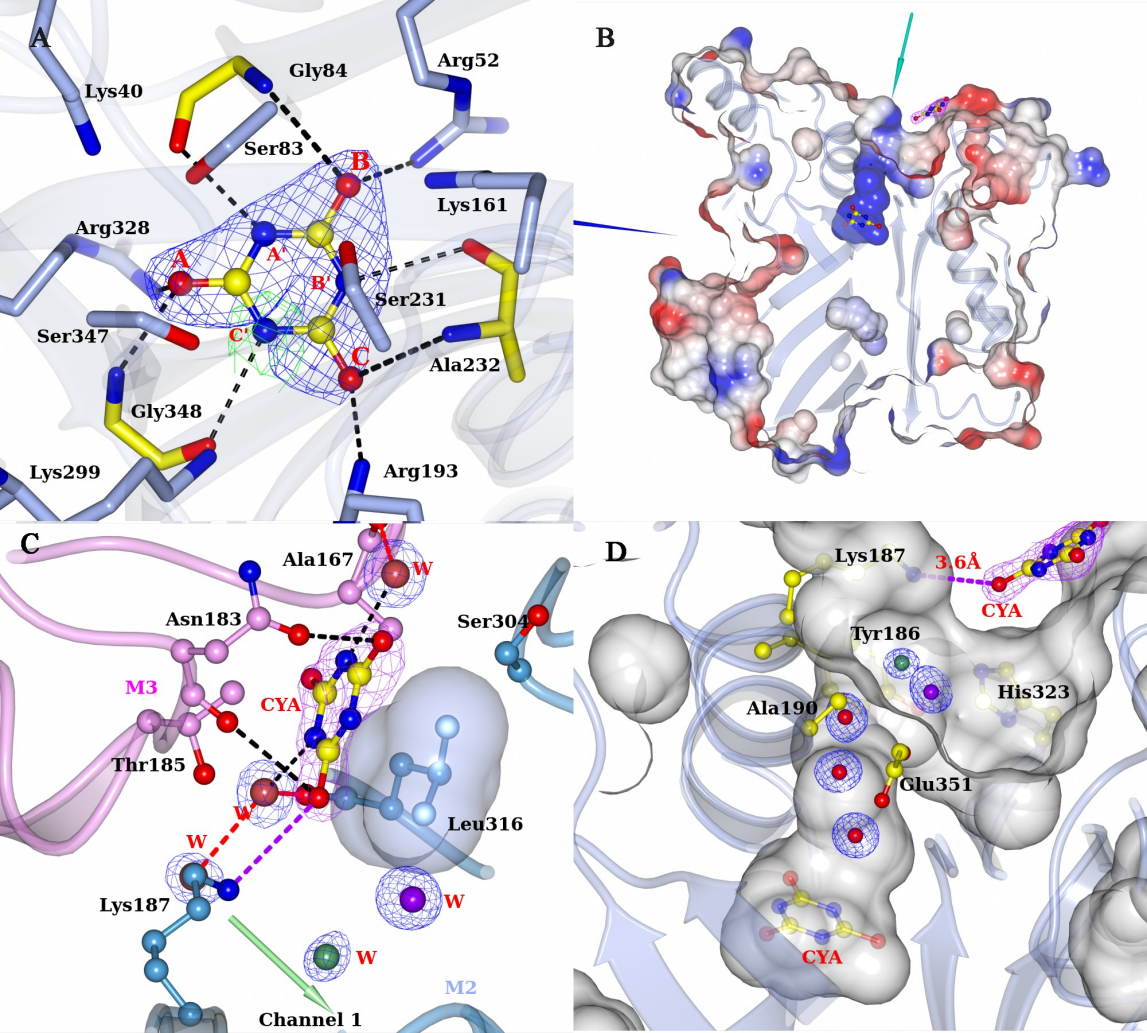
RMCAH with CYA bound. A. 2mFo-DFc simulated annealing composite omit electron density map contoured at 1.0 σ level depicted in blue mesh, and mFo-DFc omit map with only MLA modeled in contoured at 3.0 σ level depicted in green mesh. As the strong residual density shows that there should be at least partial occupancy of CYA in the active site of the monomer M2, a CYA is placed in the map to show its existence. B. Cut-away surface representation showing two channels to the active site, indicated by green and blue arrows. One cyanuric acid molecule binds at the entry of the 1^st^ channel. C. Interactions formed between CYA and two protein monomers at the entry of the ingress channel. D. A string of water molecules lined up in the ingress channel. The size of the substrate can be seen larger than the diameter of the channel at the narrowest point. All maps in B, C, D are the mFo-DFc simulated annealing composite omit map shown in pink or blue mesh.

Each RMCAH monomer shows two channels that connect the deeply buried active site to the exterior of the protein. As noted for the equivalent channels observed in the other CAH structures previously determined[8, 9, 30] these channels could serve as paths for the substrate/product to the active site. The two channels show contrasting electrostatic properties (Fig 5A). The first one, ~15 Å deep from the surface of the monomer, is formed along the pseudo-three-fold axis of the monomer (shown by the cyan arrow in Fig 5A) and is filled with a string of water molecules. The channel is lined by 20 residues with only five of these residues being at the mouth of the substrate tunnel, namely Tyr186, Lys187, Ala190, His323, and Glu351. Notably, the width at the narrowest point of this channel (~ 4 Å) is smaller than the cyanuric acid substrate (diameter of ~ 6.5 Å). The second channel (indicated by the blue arrow in Figs 5A and 6B), on the other hand, is blocked by the side chain of Lys161. Thus, the question of how the substrate gains access the active site needs to be addressed.

MLA is required for RMCAH crystallization, however its tight binding to the active site hinders further structural studies of the enzyme’s catalytic mechanisms. To obtain an apo RMCAH structure, we conducted a series of crystal-soaking experiments, reducing MLA concentration in steps until no MLA was present in the soaking buffer. The crystal structure shows that while MLA molecules still remain bound in three monomers (monomers M1, M2, M3) of the tetramer, one (monomer M4) was depleted of MLA with only very weak electron density left (named “APO” monomer). Superposition of the APO monomer onto the MLA bound structure reveals that the APO monomer adopts an “open” conformation. Domains 1 and 3 in the open conformation superpose onto corresponding domains in the closed (MLA-bound) conformation very well, while domain 2 shows a rotation by 17 degrees around an axis passing through the interface between domains 2 and 3 (Fig 5B and 5C, S1 Video), calculated using pdbSUM [31], rendering the proposed substrate entry channel more open. The γO of Ser231 and Ser347 moved away from the substrate binding site and are pointing to the inside of their respective domains. Of the three Ser-Lys dyad pair interactions observed in the closed conformation, only Ser83-Lys40 hydrogen bond is kept in the open conformation. Lys161 side chain adopts an extended conformation, forming a direct or water mediated hydrogen bond with carbonyl oxygen and amide nitrogen atoms of Phe80, respectively, rendering the second channel open. In the RMCAH crystal, monomer M4 makes the least extensive crystal contacts, which likely enabled the conversion to the open conformation *in crystallo*. The closed and open structures show little difference in crystal contacts, explaining the ready conversion between these alternative conformations.

### Calcium ion and ion switch

Addition of Mg^2+^ or Ca^2+^ had been demonstrated to modestly enhance the CAH enzymatic activities by about 20%[13] and CaCl_2_ was found to be essential for obtaining high-quality RMCAH crystals. The electron density map clearly reveals one metal ion bound in domain 3 of each monomer in the MLA bound crystals, interacting with the δO of Glu301 carboxyl group, one water molecule, and the carbonyl oxygen atoms of Ala350, Gln353, Pro355, and Gly358. The octahedral coordination is consistent with the presence of Ca^2+^ in this site (Fig 5D). However, in the monomer M4 adopting the open conformation, the electron density for the calcium ion became weaker and the distances between the coordinating atoms and the Ca^2+^ ion (listed in S4 Table) became longer in the refined model, suggesting that the site is less well occupied.

Sequence alignment shows that the Ca^2+^ ion coordination loop with closely flanking sequences, ^347^SGGAEHQGPDGGG^359^, is well conserved in the cyanuric acid hydrolase/barbiturase protein family. The overall conformations of domain 3 in the open and closed monomers remain unchanged (r.m.s.d. of 0.4 Å) except for the short segment Ser347-Gly348-Gly349-Ala350-Glu351-His352-Gln353 (containing the C-terminus of the substrate-binding β-strand and the N-terminus of the Ca^2+^ coordination loop), which shows significant displacement. This segment contains three functionally important amino acid residues: Ser347 that is one of the three active site serines, Gly348 that helps position the substrate by forming two hydrogen bonds with substrate carboxyl oxygen (position A) and amide NH (position C’), and Ala350 that coordinates Ca^2+^ using main chain carbonyl oxygen atom. The side chains of Glu351-His352-Gln353 form extensive hydrogen-bonding interactions with residues of domain 2 (Fig 5E). The side chain of Glu351 forms two salt bridges with Arg193 guanidinium group, which in turn interacts with substrate carbonyl oxygen atom at position C. The imidazole ring of His352 forms two hydrogen bonds with the backbone N atom of Cys218 and O atom of Leu223. The carbamoyl group N and O atoms of Gln353 form hydrogen bonds with side chain γO atom and main chain NH of Thr230, respectively. The segment of Ser347-Gly348-Gly349-Ala350 does not interact with domain 2 directly but has large displacement (Fig 5E, S2 Fig). The coordinated movement between the Ca^2+^ binding loop, the flanking substrate-binding β-strand, and domain 2 could suggest the role of Ca^2+^ in stabilizing the RMCAH closed conformation and substrate binding. Interestingly, all previous crystallographic work of CAH showed the presence of divalent ions at this position [8, 9] while functional significance of the metal-binding was unclear.

To further explore the open/closed conformations and dynamic properties of RMCAH, the APO crystals were soaked with increasing MLA concentrations in the soaking buffer until the original crystallization precipitant concentration was reached. The resulting structure (MLA-R in Table 1) is essentially identical to the initial MLA bound structure, with one copy of MLA bound at the active site of each monomer in the tetrameter and each monomer adopts closed conformation. Furthermore, the weakened electron density of Ca^2+^ in monomer M4 is recovered to the original peak height. The monomer M4 in the original MLA bound and regenerated MLA bound from APO crystals have an r.m.s.d. of only 0.24 Å for all Cα atoms. However, the domain 2 of monomer M4 shows increased average B-factors (S2 Table). The reversible enzyme conformational changes and Ca^2+^ binding suggests that Ca^2+^-binding may be coupled to the catalytic cycle of CAH. Although we did not find Ca^2+^ to be essential for the catalytic activity of RMCAH, a point mutation of one of the Ca^2+^-coordinating residues Glu301 (E301A) reduced the stability of RMCAH (S3 Fig), suggesting possible structural importance of the metal ion binding.

### Ligand bound structures

#### RMCAH/Cyanuric acid (CYA) complex structure

The RMCAH/CYA complex structure was obtained by soaking the APO crystals with buffer containing CYA at pH 5 and 4°C, under which condition the CAH catalytic activity is negligible. The structure shows that the monomer M4 remained unchanged in the open conformation. Refinement with only malonate at the active sites reveals residual mFo-DFc density in the active site of monomer M2 (Fig 6A) and a weaker density in M3 suggesting that there is likely a certain amount of cyanuric acid bound. Modeling CYA in the active site of monomer M2 and subsequent refinement gave a partial occupancy of 0.3. The significant occupancy of non-hydrolyzed CYA in the active site of M2 might correlate with M2 taking a slightly more open conformation than monomers M1 and M3 (S4A Fig) and a potentially reduced catalytic rate in the crystal. The details of the interactions between cyanuric acid and the active site residues are shown in Fig 6A.

Aside from binding at the active site, another cyanuric acid molecule is found bound to the RMCAH surface near the entrance of the first channel of the monomer M2 (Fig 6B), which supports the channel's function as a substrate entrance and provides insights as to how the substrate accesses the channel. One face of this cyanuric acid molecule stacks on the Leu316 side chain, while the other face is stabilized through hydrophobic interactions with the Thr185 side chain and hydrogen bonding interactions to OD1 and O atoms of Asn183 from the neighboring molecule (Monomer M3) within the tetramer (Fig 6C). A string of water molecules lines up in the channel (Fig 6D). We had observed a barbiturate molecule bound similarly near the entrance of the channel in the ACAH structure[9]. These observations suggest that the residues near the channel entrance and subunit interface of CAH play an active role in attracting the substrate into the enzyme active site.

#### RMCAH/intermediate analog

**Fig 7:**
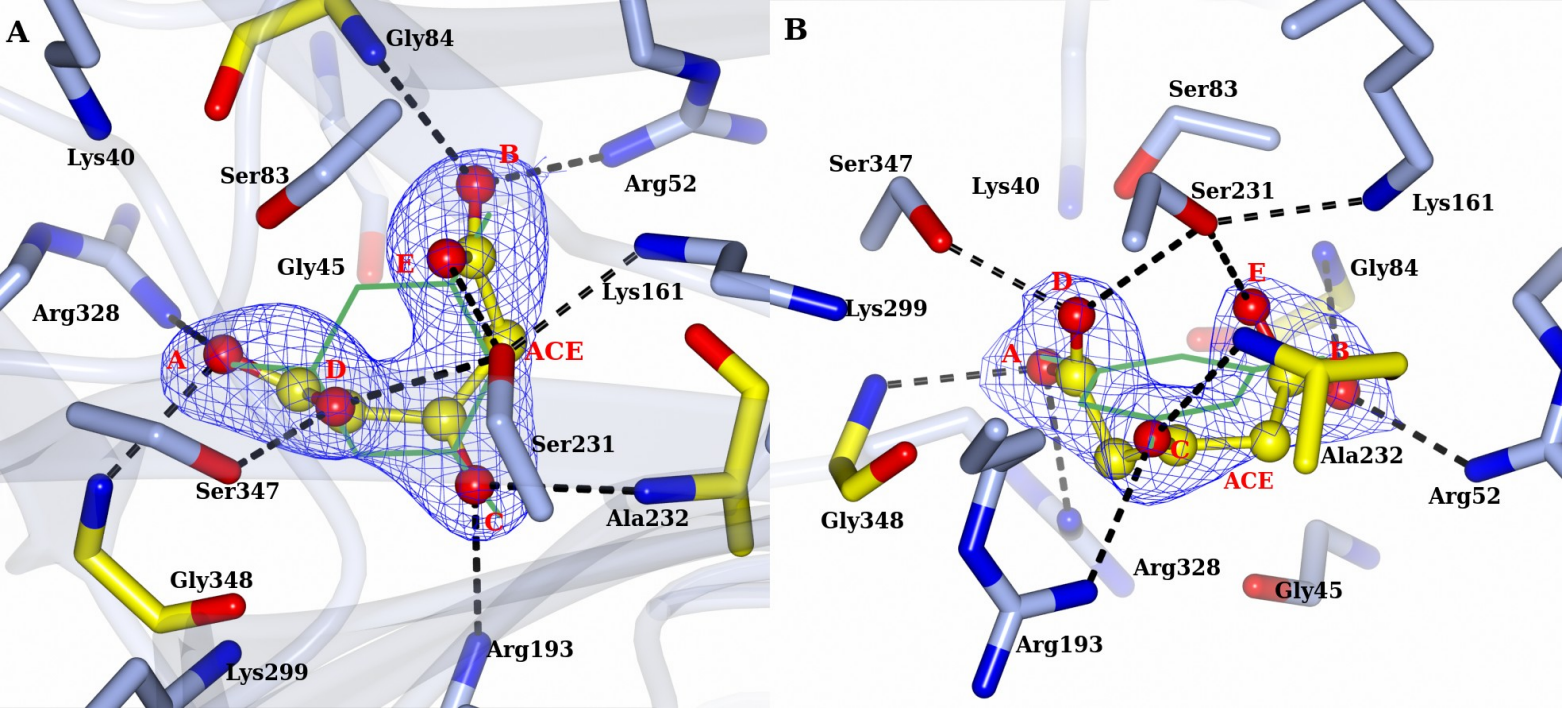
RMCAH with 1,3-acetonedicarboxylic acid (ACE) bound. A. Top-view of 2mFo-DFc electron density map contoured at 1.0 σ level, depicted in blue mesh on ACE. ACE is shown in ball-and-stick. Hydrogen bonds are indicated by the black dotted lines. A reference barbituric acid molecule from the RMCAH/BAR structure (depicted in green lines) is overlaid to show the substrate plain and the relative location of ACE atoms. B. Side-view of ACE bound in the active site.

Since the ring-opened intermediate carboxybiuret decomposes within the order of minutes, several more stable chemicals mimicking carboxybiuret were tested to probe RMCAH structure with the reaction intermediate bound. The compounds used included 1,3-acetonedicarboxylic acid, 1-nitrobiuret, and 5-azacytosine. Only the 1,3-acetonedicarboxylic acid (hereafter referred to as ACE, Fig 1F) bound structure was obtained. Three out of a total of five oxygen atoms (Fig 7A and 7B), one from each of the two carboxyl groups and one from the carbonyl group in the middle of the molecule, roughly stayed in the substrate plane. They occupy almost the same positions as the three carbonyl oxygens of cyanuric acid (position A, B, C) with the same sets of interactions as those observed for CYA (Fig 7A). The oxygen atom at position B is slightly below the substrate plane (~0.5 Å) and the carbonyl oxygen of Gly45 accordingly moved away from the substrate plane to accommodate this off-plane oxygen atom at position B (Fig 7B). One of the remaining carboxyl oxygen atoms OAF (Fig 1F) is out of the plane due to the rotation of carboxyl group around the CAE-CAD bond and occupies the D position as seen in the MLA bound structure, forming hydrogen-bonding interactions with all the γO atoms of three serine residues at the active site (Ser83, 3.0 Å; Ser231, 2.9 Å; Ser347, 3.1 Å). The oxygen atom occupying the D position led to expansion of the triangle formed by the three Ser γO atoms as in the RMCAH/MLA complex. Similarly, the oxygen atom OAJ (Fig 1F) of the other carboxyl group swings out of the plane and occupies position E, forming hydrogen-bonding interactions with γO atoms of Ser83 (3.0 Å) and Ser231 (2.6 Å). εN of Lys161 is 3.4 Å away to OAJ. Therefore, in addition to its role in deprotonating Ser231 for nucleophilic attack on one of the carbonyl groups of cyanuric acid, Lys161 could also play a role in neutralizing the developing negative charge during the ring-opening reaction. Superposition of the four monomers shows variation of the domain 2 positions, with the molecules M2 and M4 taking a slightly more open conformation (S4B Fig).

#### RMCAH/Barbiturate complex structure

**Fig 8:**
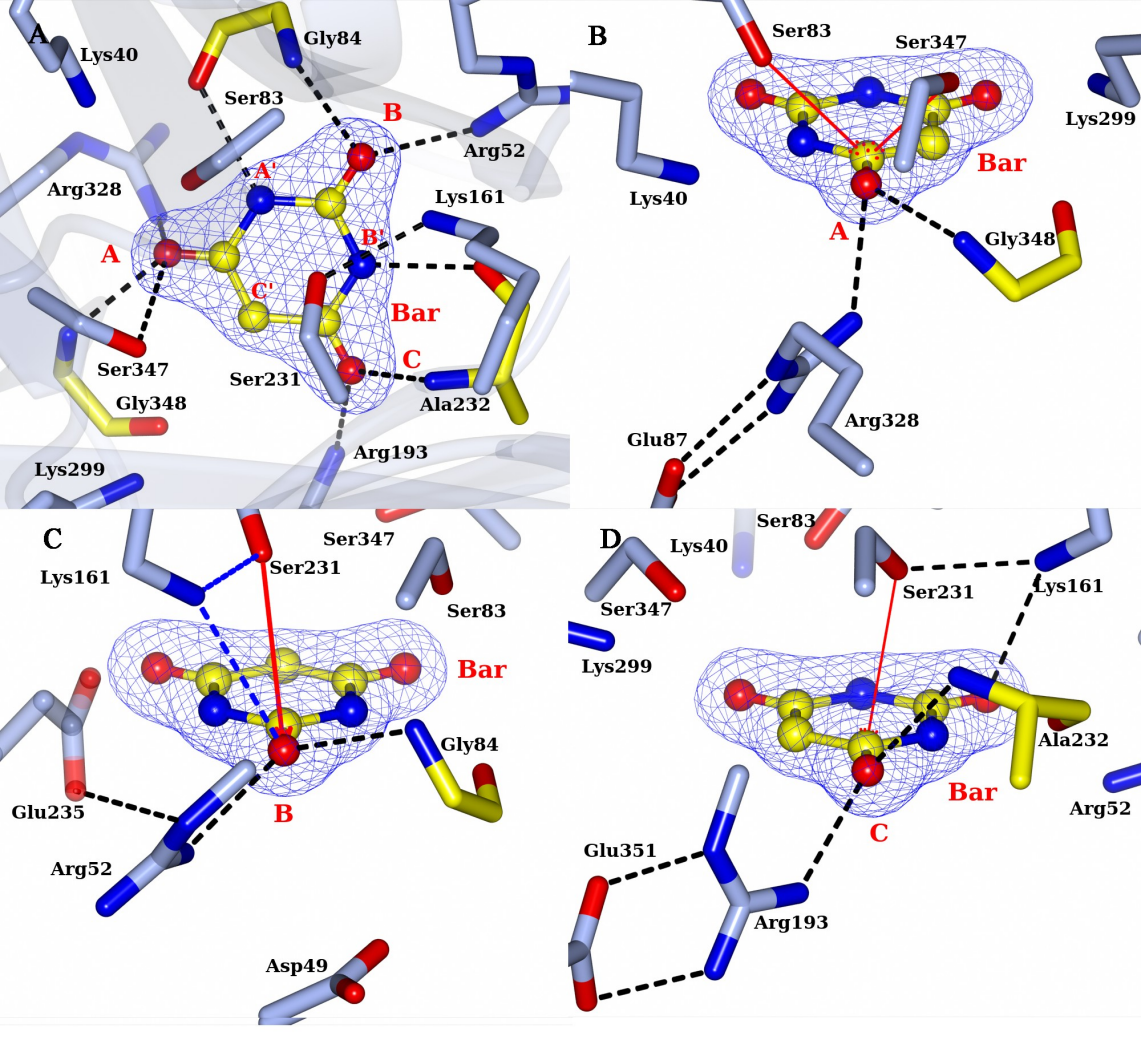
RMCAH with barbituric acid (BAR) bound and the detailed environment for each of the three carbonyl oxygen atoms. A. 2mFo-DFc electron density map contoured at 1.5σ level, depicted in blue mesh. B. Site A, the αFL for Ser83 and Ser347 are shown in red arrows. The hydrogen bonding interactions for the carbonyl group are shown by dashed lines. C. Site B. Only Ser231 is close enough for initiating the reaction. The distance/αBD/αFL of Ser231 to the carbonyl group carbon of BAR are 3.4Å/110°/5°. Side chain of Glu235 forms one hydrogen bond with the side chain of Arg52. D. Site C. the distance/αBD/αFL of Ser231 to the carbonyl group carbon of BAR are 3.3Å/106°/7°. Side chain of Arg193 forms two hydrogen bonding interactions with side chain of Glu351, which is in the Ca^2+^ coordination loop.

By soaking the APO RMCAH crystal with the cyanuric acid analog and a competitive inhibitor barbiturate (BAR, Fig 1B and 1D), we obtained a BAR-bound structure with all four monomers in the closed conformation. The positioning of the barbiturate (BAR) in the active site is similar to that observed in the previously published BAR-bound structure of ACAH (4NQ3). However, the significantly improved resolution, along with structures with other ligands reported in this study, enables a more thorough examination of the complex as well as comparative analysis. Both planar and non-planar BAR were used in the refinement, and the planar BAR fit in the electron density better. Fig 8A shows the top-view of simulated-annealing composite omit electron density map contoured at 1.0 σ level. Even though the BAR electron density shows nearly three-fold symmetry, the bound BAR in the active site is likely orientated in a specific configuration based on its positioning relative to the surrounding protein residues. At A’ and B’ positions, the distance between a ring nitrogen atom of BAR and the backbone carbonyl oxygen atom of Gly84 and Ala232 from RMCAH are 2.8 and 3.0 Å, respectively, while at the C’ position, the distance between the α-carbon atom of BAR and Gly348 is 3.2 Å. Viewed from top of BAR along its pseudo three-fold axis, Ser83 and Ser347 γOs are both exterior to the ring, while Ser231 γO is directly above the BAR ring and points to the mid-point of BAR carbon atoms of carbonyl group at B and C. Each carbonyl oxygen atom of the inhibitor at the sites A, B, and C forms hydrogen-bonding interactions with a main chain amide nitrogen atom and an arginine side chain. Fig 8B, 8C and 8D show the views focusing on each of the BAR carbonyl oxygens. Of note, the four monomers (M1~M4) within the RMCAH tetramer showed varied exchange rates for BAR in the active site (S3 Table, BAR-a, BAR-b), with the more flexible M4 and M3 (S2 Table, B-factors reflecting flexibility: M1<M2<M3<M4) showing higher rates. The observation supports that the dynamic properties of the monomers are important for the intake of the ligands.

Fig 8B show the detailed interactions at site A. The distance/Bürgi–Dunitz angle[32] (αBD)/Flippin–Lodge angle[33] (αFL) of Ser83 γO to the nearest carbonyl group of BAR are 3.6Å/80°/35°, and that of Ser347 are 3.4Å/65°/45°. The main chain N atom of Gly348 is in the substrate plane and has a distance of 2.7 Å to the carbonyl oxygen (Fig 8B). Arg328 side chain NH1 atom is almost directly under BAR carbonyl oxygen at A site with a distance of 2.8 Å. The Arg328 side chain position is stabilized by two hydrogen bonds with the side chain of Glu87, which in turn forms a hydrogen bond with the side chain of Arg325. At the B site, the distance/αBD/αFL of Ser231 γO to carbonyl group carbon of BAR are 3.4Å/110°/5°. The main chain N atom of Gly84 is pointed at a lone-pair (*sp*^*2*^) electron orbital of carbonyl oxygen and slightly above the substrate plane, with an angle of ~20° off the plane and has a distance of 3.0 Å (Fig 8C). Arg52 side chain NH1 atom is at the direction of the other lone-pair electron orbital of the carbonyl oxygen and slightly below the plane, with an angle of ~60° and a distance of 2.8 Å. The NH atom of Arg52 forms a hydrogen bond with Glu235 side chain carboxyl oxygen. There is no other negatively charged group within 4 Å of the Arg52 guanidinium group. At the C site, the distance/αBD/αFL of Ser231 γO to carbonyl group carbon of BAR are 3.3Å/106°/7° (Fig 8D). The main chain N atom of Ala232 is slightly above the substrate plane, with an angle ~20° and has a distance of 3.0 Å. Arg193 side chain NH1 atom is at the direction of a *sp*^*2*^ electron orbital of carbonyl oxygen and slightly below the plane, with an angle of ~65° and a distance of 2.8 Å. The NH atom forms a hydrogen bond with Glu351 side chain carboxyl oxygen. Taken together, the atomic configurations around the three carbonyl oxygen atoms of the substrate/inhibitor are quite different.

### Conformational dynamics in solution

**Fig 9:**
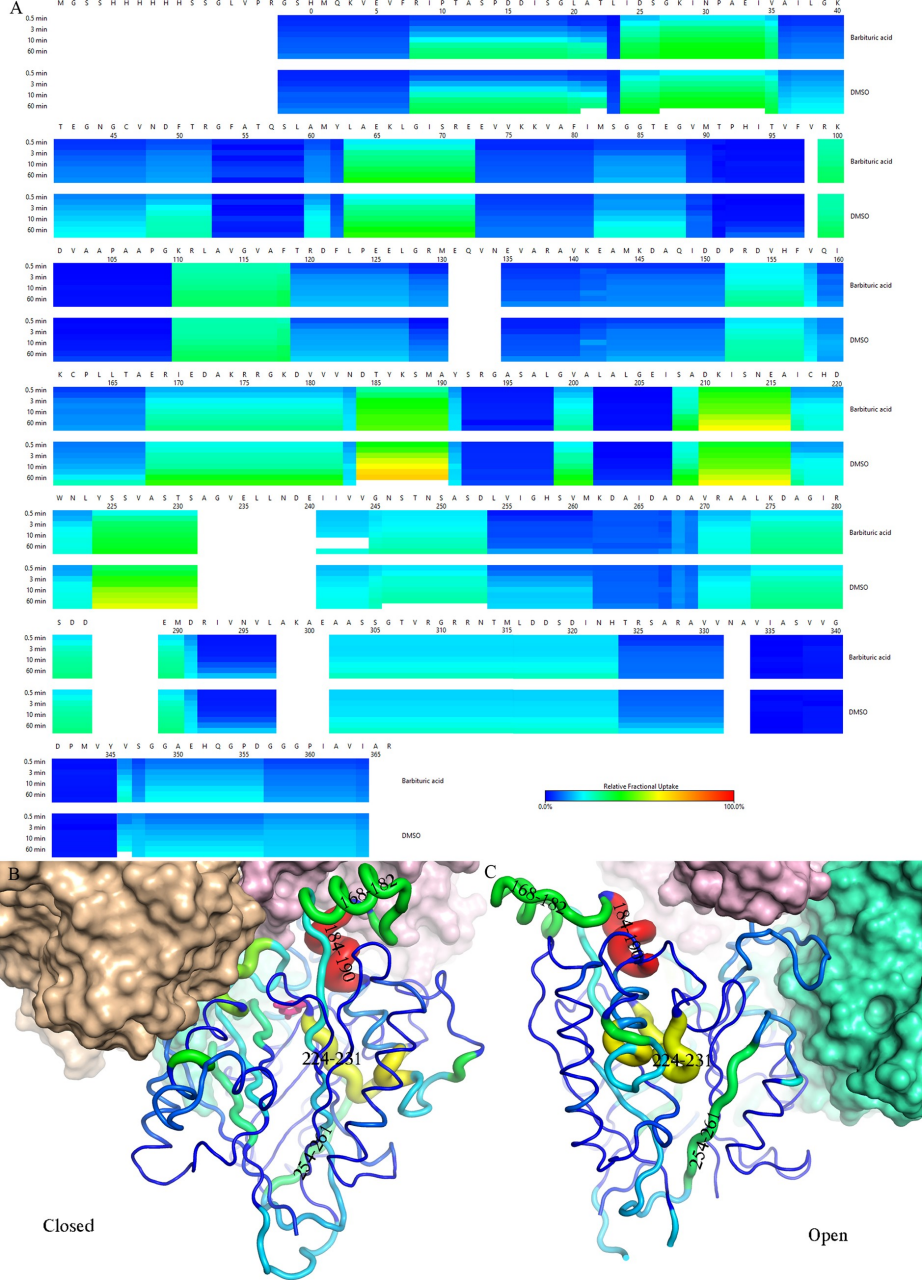
Hydrogen-deuterium exchange mass-spectroscopy (HDX-MS). A. Heat map showing the relative fractional deuterium uptake at indicated time points for proteolytic fragments of apo RMCAH (DMSO control, lower strip) and RMCAH/BAR complex (upper strip). Lowest exchange rate is depicted in blue and highest in red. Residues not detected in MS are shown in white. B, C. Tube representation of the difference in HDX rate between RMCAH and RMCAH/BAR, mapped on the closed BAR-bound (B) and apo (C) structures of RMCAH. Smaller differences are depicted in thin tube and blue color, while larger differences are in thick tube and red color. Note that the color-coding in (B) and (C) has a different meaning from that in (A).

The reversible opening/closure of domain 2 and ligand exchange in *crystallo* demonstrated the importance of conformational dynamics in substrate-binding. To probe the protein dynamics in solution, we performed hydrogen-deuterium exchange mass-spectrometry (HDX-MS) and compared the RMCAH/BAR complex with ligand-free RMCAH. In the presence of saturating (10 μM) BAR[34, 35], which stabilizes the closed conformation of RMCAH in crystals, we observed reduced deuterium uptake rate for a number of regions of RMCAH (Fig 9). The most significantly affected residues are located in domain 2, buried inside the RMCAH monomer or at a subunit interface within the tetramer in the closed conformation (D184-A190, Y224-S231), and in domain 3 at the interface with domain 2 (L254-M261). Other residues that were protected in the presence of BAR also map to interior of the RMCAH monomer. Thus, the HDX-MS data support that the domain 2 is flexible in solution and it closes down upon substrate/inhibitor binding in the active site.

To further probe functional importance of the domain 2 flexibility, we generated a double-mutant enzyme ‘RMCAH-CC’ (G53C/E235C) in which Gly53 and Glu253 from domain 1 and 2, respectively, have been changed to Cys. We confirmed that these two cysteines introduced near the domain1/2 interface can form a disulfide bond, by determining a crystal structure of RMCAH with several additional mutations C46S/C162A/C218V/G53C/E235C. This structure showed the domain 2 locked in a partially open conformation via the disulfide linkage (S5A Fig). Notably, RMCAH-CC showed higher activity in the presence of 10 mM DTT than in the absence of DTT. This was in contrast to wild-type RMCAH that was slightly inhibited by the high concentration of DTT (S5B and S5C Fig) possibly due to an adduct formation involving cysteine residues near the active site. These data corroborate that the flexibility of domain-2 is important for the catalytic activity of CAH enzyme.

## Discussion

### Multiple conformations/enzyme dynamics, substrate binding and metal ions

In the present study we have determined a series of crystal structures of RMCAH by replacing ligands *in crystallo* through sequential soaking. A comparison between the RMCAH structures obtained with different ligands in the active site revealed an unexpected conformational flexibility; the positions of domain 1 and domain 3 remain un-changed, while domain 2 has varying degrees of displacement (Fig 5B and 5C, S4 Fig). Two distinct conformations of the CAH monomer corresponding to open *vs*. closed states were observed, which differed by a rotation of domain 2 by 17 degree (Fig 5B and 5C). The large movement of domain 2 is consistent with the quaternary architecture of tetrameric CAH, in which the domains 1 and 3 of each monomer form the tetramer core while domain 2 faces outside of the tetramer and is involved in fewer interactions with other monomers in the tetramer. The wide open conformation of the CAH monomer observed in the APO crystal, and the ready interconversion between the open and closed conformation coupled with ligand-binding demonstrate a dynamic property of RMCAH and suggest its role in substrate binding and catalysis. The active site of CAH is deeply buried, and the diameter of the channel for substrate ingress in the closed conformation is actually smaller than the size of the substrate. The narrow channel is occupied by a string of water molecules lining up in the channel. The dynamics of the enzyme shown in the present study demonstrates a possible means for the substrate to reach the active site through this channel. The HDX-MS experiment corroborated the crystallographically observed movement of domain 2 in solution. Along with the conformation changes through displacement of domain 2, the flexible segment Ser347-Gly348-Gly349-Ala350-Glu351-His352-Gln353 in domain 3, interacting with substrate at one end and Ca^2+^ at the other end and undergoes a coordinated movement with the domain 2, couples the metal ion coordination to substrate-binding. In the “open” conformation, the Ca^2+^ coordinating loop becomes less compact, the Ca^2+^ could dissociate, and the β-strand 3 (harboring the substrate-interacting residues Ser347 and Ala348) of domain 3 have large displacement from the substrate binding pocket. The binding of Ca^2+^ makes the metal-coordinating loop more compact and stabilizes the β-strand 3 in the conformation more favorable for substrate binding. Thus, despite the symmetry of the cyanuric acid and the corresponding overall three-fold symmetry of the enzyme fold and its active site configuration, the three isostructural domains have distinct and non-symmetrical roles during substrate binding.

### The ring-opening reaction and catalytic serine

The active site is formed at the end of β-strands 3 from each of the three domains. Three Ser residues, Ser83, Ser231 and Ser347, make up a nearly equilateral triangle. As observed for ACAH and AtzD[8, 9], each of the three serine γO atoms is in close proximity to a lysine residue (Lys40, Lys161 and Lys299), generating three pairs of Ser-Lys dyads each from one of the three isostructural domains from the same monomer. The Ser-Lys dyad paired with a Arg residue, which contributes to substrate coordination, are configured similarly in each domain. In our previously reported crystal structure of ACAH, it was proposed that the serine in domain 2 (Ser231 in RMCAH and Ser226 in ACAH) is the likely nucleophile, while the other two serines (Ser83, Ser347 and Ser79, Ser333 in RMCAH and ACAH respectively) are involved in substrate activation and orientation of the substrate in the pseudo three-fold symmetric active site. Our present study with much improved resolution adds important information about the catalytic role of Ser231. It has been described that Bürgi–Dunitz angle (αBD) and Flippin–Lodge angle (αFL) for nucleophilic attack at a carbonyl carbon concentrated in a rather narrow range in enzyme-catalyzed reactions[32, 33, 36]. The present RMCAH/BAR structure refined at 1.88 Å resolution shows that the αBD/αFL angles (110° and 5°) and the distance (3.4 Å) between the Ser231 γO and a substrate carbonyl group are well within favorable ranges for initializing the nucleophilic addition reaction, in contrast to unfavorable parameters for the other serine residues (Ser83 80°/35°/3.6Å, Ser347 45°/65°/3.6Å). Previously reported ACAH and AtzD structures had limited resolution and were obtained only with three-fold or pseudo-three-fold symmetrical planar ligands bound in the active site, which highlighted the symmetrical aspects of the active site and only showed the atom position of the ring compounds within the substrate plane. The present study has complex structures with MLA and ACE, which lack the three-fold symmetry, bound in the active site and thus makes it possible to probe the micro-heterogeneity of the RMCAH active site and show the possible off-plane atom locations of the open-ring intermediate. Based only on the distance/αBD/αFL, nucleophilic attack by Ser231 γO on the carbonyl group at site C is equally plausible as that at site B. However, as discussed below, our MLA/RMCAH and ACE/RMCAH complex structures suggest that the reaction intermediates are likely better stabilized in the active site when the reaction starts by a nucleophilic attack on the carbonyl group occupying the site B.

Both MLA and ACE have two of their carboxyl groups bound at the A and B sites exclusively, which suggests that the A and B sites of the RMCAH active site are well capable of accommodating negatively charged groups as oxyanion holes. Analysis of the environments for each active site arginine residue supports experimental results. The three Ser-Lys-Arg clusters are contained in a rather small volume (157 Å^3^, calculated by CASTp server[37]) and form the active site pocket of RMCAH to accommodate cyanuric acid, which itself occupies a space of 94 Å^3^. The opening of the six-membered ring during the reaction and following the rule of αBD will require that the reaction intermediate and/or enzyme undergo significant conformational changes[32]. At A site, the Arg328 NH1 atom is almost directly underneath the substrate carbonyl group and forms a tetrahedral cage with three γOs of Ser83, Ser231 and Ser347, limiting the mobility of carboxylate or amide group in the cage. Superposition of 4BVQ, RMCAH/MLA and RMCAH/ACE structures show that two oxygen atoms from the phosphate in 4BVQ or those from a carboxylate in RMCAH/MLA and RMCAH/ACE occupy A and D positions with little positional variations. However, two oxygen atoms from the other carboxylate in RMCAH/MLA and RMCAH/ACE show significant positional variation at B site: The carboxyl oxygen atom of MLA is ~1Å above the substrate plane while the oxygen atom of ACE is ~0.5 Å below the substrate plane. Regardless of the positional variation of B site oxygen atoms, the interactions are still maintained between the ligands and the enzyme. These positional variations could reflect that the ring-opening reaction initialized at B site better meets the spatial requirement for the *sp*^2^ to *sp*^3^ orbital conversion than at A site during the catalysis. Therefore, our current study suggests that the ring-opening reaction would be preferably initialized by the Ser231-Lys161 dyad at B site. This is consistent with the strict conservation of Ser231-Lys161 and Arg52 surrounding the B site while other two Ser-Lys-Arg triads have some variations.

### RMCAH/intermediate model and enzyme catalytic mechanism

**Fig 10:**
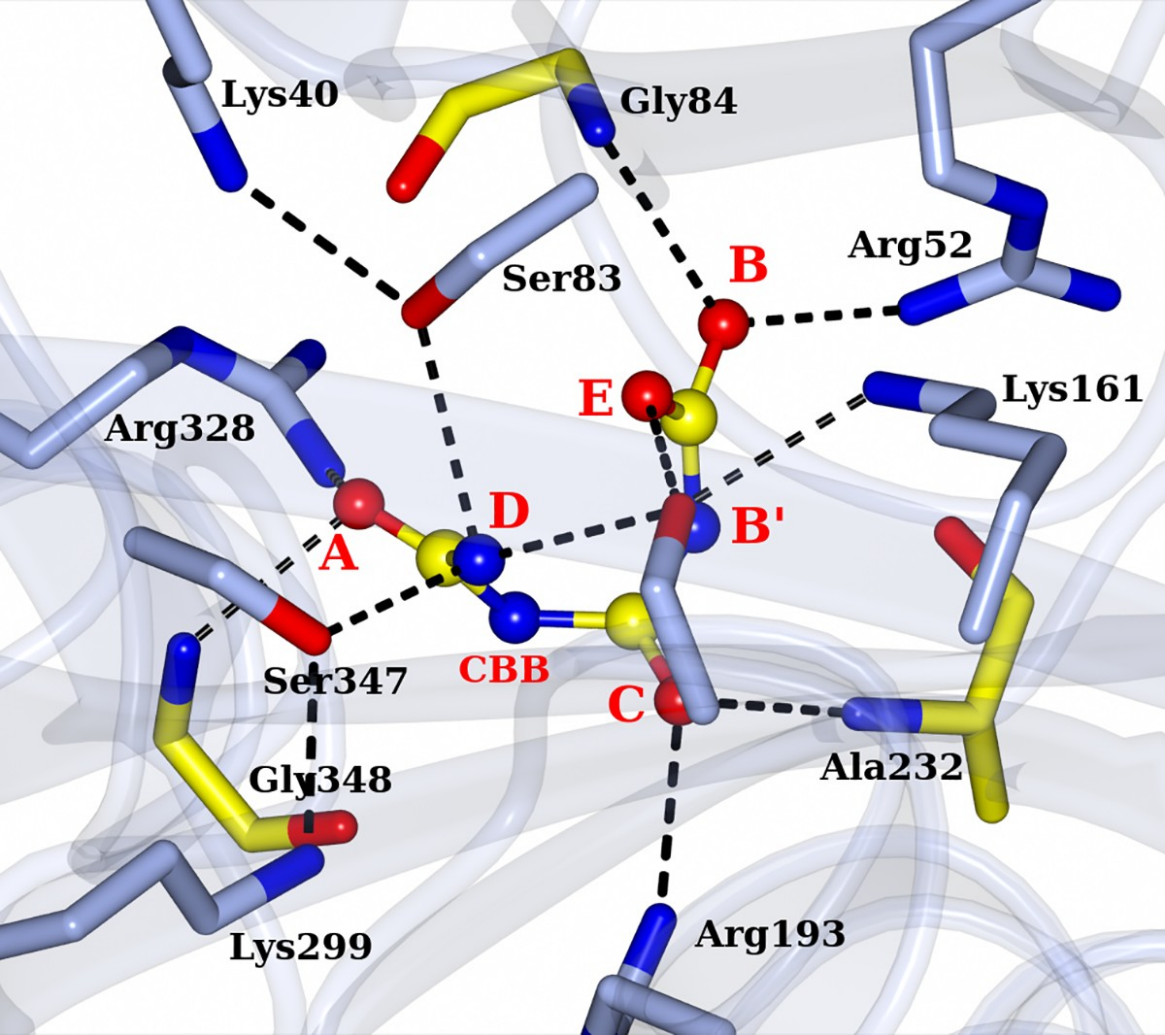
Atomic model for the reaction intermediate, carboxybiuret, bound in the active site of RMCAH. The model is based on the crystal structure of the RMCAH/ACE complex shown in Fig 7 and the active site properties revealed by other RMCAH/ligand structures.

Based on the RMCAH/ACE complex structure, we constructed a model of the RMCAH/carboxybiuret complex (Fig 1G, Fig 10). Carboxybiuret in the model, which has the same overall shape as ACE but has amide nitrogens replacing the two methylene carbons and an amino group replacing one of the carboxyl oxygens of ACE, would fit in the active site with a set of additional hydrogen bonding interactions. The O atom at one end of carboxybiuret fits in the A site, whereas the adjacent amino group is above the substrate plane and occupies the D site at the middle of three active site serine γOs. The neighboring amide group occupies the C’ site. The central carbonyl oxygen atom occupies the C position. A carboxylate oxygen on the other end of carboxybiuret interacts with the N atom of Gly232 and NH1 of Arg52 at the B site. Based on this configuration, the catalytic mechanism of CAH can be inferred: γO atom of Ser231 performs a nucleophilic attack on the carbonyl carbon atom of cyanuric acid directly bonded to the oxygen atom occupying the B site, forming a ring-opened acyl-enzyme covalent intermediate. Owing to the clash immediately after the ring-opening, the amino group needs to move out of the substrate plane. The amino group positions its N atom at the center of the triangle formed by the γO atoms Ser83, Ser231 and Ser347 (D site). Hydrolysis of the acyl-enzyme intermediate generates a carboxyl group, and one of its oxygen atoms lies above the substrate plane (E site). The flexible B site is suited for accommodating the changing structures of the reaction intermediates. If Ser83 were to perform the nucleophilic attack, it could only target carbonyl carbon of cyanuric acid directly bonded to the oxygen atom occupying the A site. Such reaction might not proceed, as the lack of flexibility of the surrounding residues at this site precludes the subsequent *sp*^*2*^/*sp*^*3*^ conversion.

The thermostable MCAH is currently used commercially for degrading cyanuric acid. Our high-resolution structures of RMCAH provide a blueprint for engineering this enzyme for bioremediation of a water disinfection by-product. For bioremediation applications, a major impediment to the use of CAH is the sensitivity of the enzyme to hypochlorite that is present in pools and spas needing water remediation. Preliminary studies have shown that key active sites residues are not oxidized by hypochlorite. Thus, the present structures can be used to map out strategic replacements of oxidizable residues by non-sensitive amino acids without blocking substrate turnover or affecting essential dynamics of the enzyme, which will allow practical use of CAH immobilized in water filtration media.

## Supporting information

S1 Table CAH structures previously reported. (DOCX)

S2 Table Average B-factors for each domain of the four monomers (M1 ~ M4) in RMCAH tetramer. (DOCX)

S3 Table Distances between active site serine γO atoms in different structures/domains. (DOCX)

S4 Table Calcium coordination in the present structures and magnesium coordination in the AtzD structure reported earlier. (DOCX)

S1 Fig Comparison of WT CAH and RMCAH activities. (PDF)

S2 Fig Domain structures of RMCAH. (PDF)

S3 Fig Reduced stability of Ca^2+^ binding variant E301A. (PDF)

S4 Fig Comparison of the structures of individual monomers in the RMCAH-ligand complexes. (PDF)

S5 Fig G53C/E235C disulfide-crosslinking. (PDF)

S1 Video A morphing movie showing the domain-2 movement between the ligand-bound closed conformation and the open APO conformation of RMCAH. (MOV)

## Abbreviations

CAH: Cyanuric Acid Hydrolase
RMCAH: Re-engineered *Moorella* CAH
BAR: barbiturate
ACE: 1,3-acetonedicarboxylic acid
MLA: malonate
αBD: Bürgi–Dunitz angle
αFL: Flippin–Lodge angle
