# Supplementary material for: Crystal structures of Moorella thermoacetica cyanuric acid hydrolase reveal conformational flexibility and asymmetry important for catalysis
Source: PLoS One. 2019 Jun 10;14(6):e0216979. doi: 10.1371/journal.pone.0216979 (PMC6557486; doi:10.1371/journal.pone.0216979)
Supplement: S1 Table — (DOCX) [file pone.0216979.s001.docx]

**S1 Table****.** **CAH structures previously reported**

| PDB code | Reso. (Å) | Ligand | Ligand geometry | Ligand Symmetry |
| --- | --- | --- | --- | --- |
| 4BVQ^I^ | 1.90 | Phosphate ion | tetrahedral |  |
| 4BVR^II^ | 2.58 | Cyanuric acid | Planar normal triangle | 3-fold |
| 4BVS^II^ | 2.60 | Melamine | Planar normal triangle | 3-fold |
| 4BVT^II^ | 3.1 | Barbituric acid | ~Planar normal triangle | Pseudo 3-fold |
| 4NQ3^II^ | 2.7 | Barbituric acid | Planar normal triangle | Pseudo 3-fold |
| 5HY1^II^ | 2.01 | Cyanuric acid | Planar normal triangle | 3-fold |
| 5HWE^III^ | 1.71 | ADA |  | Pseudo 3-fold |
| 5HXU^III^ | 1.83 | ADA |  | Pseudo 3-fold |
| 5HXZ^III^ | 2.36 | ADA |  | Pseudo 3-fold |

The structures of CAH from Pseudomonas sp. strain ADP and Azorhizobium caulinodans ORS 571 reported previously[8, 9] were classified into three groups:

I. Native structure with a phosphate ion bound to one corner of the active site.

II. Five complex structures with substrate or substrate analogues.

III. Three complex structures with buffer components. ADA: N-(2-acetamido)iminodiacetic acid.
