# Supplementary material for: Crystal structures of Moorella thermoacetica cyanuric acid hydrolase reveal conformational flexibility and asymmetry important for catalysis
Source: PLoS One. 2019 Jun 10;14(6):e0216979. doi: 10.1371/journal.pone.0216979 (PMC6557486; doi:10.1371/journal.pone.0216979)
Supplement: S2 Table — (DOCX) [file pone.0216979.s002.docx]

**S2 Table. Average B-factors (Å^2^) for each domain (Domain 1 ~ 3) of the four monomers (M1 ~ M4) in a tetramer**

|  | M1 | M2 | M3 | M4 |
| --- | --- | --- | --- | --- |
| Domain 1(MLA) | 20.04 | 19.91 | 24.18 | 33.36 |
| Domain 2 | 20.92 | 21.58 | 25.66 | 38.76 (18) |
| Domain 3 | 19.39 | 20.62 | 20.50 | 27.31 |
| Domain 1(APO) | 19.66 | 20.25 | 27.42 | 33.52 |
| Domain 2 | 21.50 | 26.51 | 30.10 | 52.35 (31) |
| Domain 3 | 20.54 | 21.23 | 23.36 | 25.61 |
| Domain 1(MLA-R) | 26.63 | 27.19 | 32.22 | 41.61 |
| Domain 2 | 29.68 | 31.13 | 35.00 | 62.23 |
| Domain 3 | 26.85 | 28.50 | 27.79 | 37.25 |
| Domain 1(CYA) | 30.43 | 31.00 | 38.91 | 50.05 |
| Domain 2 | 33.46 | 43.37 | 41.88 | 83.55 (50) |
| Domain 3 | 31.39 | 34.40 | 33.46 | 40.30 |
| Domain 1(ACE) | 35.50 | 35.72 | 38.24 | 51.01 |
| Domain 2 | 42.26 | 43.38 | 41.55 | 67.23 (25) |
| Domain 3 | 36.17 | 35.24 | 34.77 | 40.97 |
| Domain 1(BARa) | 29.46 | 30.29 | 42.10 | 55.49 |
| Domain 2 | 32.40 | 35.01 | 43.48 | 69.41 (37) |
| Domain 3 | 30.87 | 34.14 | 32.25 | 43.08 |
| Domain 1(BARb) | 33.28 | 33.81 | 45.82 | 57.58 |
| Domain 2 | 36.24 | 43.75 | 46.71 | 73.09 (36) |
| Domain 3 | 34.71 | 38.12 | 36.65 | 47.47 |

The difference in B-factor between the domain 2 of M4 (highest) and that of M1(lowest) is shown in parenthesis for each crystal structure. The APO form RMCAH, obtained by soaking the crystals in malonate-free condition, has significantly increased B-factor at domain 2 of M4 compared to the parental malonate-bound (MLA) structure.
