# Supplementary material for: Crystal structures of Moorella thermoacetica cyanuric acid hydrolase reveal conformational flexibility and asymmetry important for catalysis
Source: PLoS One. 2019 Jun 10;14(6):e0216979. doi: 10.1371/journal.pone.0216979 (PMC6557486; doi:10.1371/journal.pone.0216979)
Supplement: S3 Table — (DOCX) [file pone.0216979.s003.docx]

**S3 Table. Distances between active site serine γO atoms in different structures/domains**

| Structure | Monomer | Ligand | Ser83-Ser231 | γOs distances (Å)  Ser83-Ser347 | Ser231-Ser347 |
| --- | --- | --- | --- | --- | --- |
| RMCAH/MLA | M1 | MLA | 3.9 | 4.0 | 4.4 |
|  | M2 | MLA | 4.0 | 4.1 | 4.4 |
|  | M3 | MLA | 3.9 | 4.0 | 4.4 |
|  | M4 | MLA | 3.9 | 4.0 | 4.3 |
| “APO” | M1 | MLA | 3.9 | 4.0 | 4.3 |
|  | M2 | MLA | 3.9 | 4.1 | 4.4 |
|  | M3 | MLA | 4.0 | 4.1 | 4.5 |
|  | M4 | None | 7.8 | 4.7 | 7.2 |
| RMCAH/MLA | M1 | MLA | 3.9 | 4.0 | 4.3 |
| Re-generated | M2 | MLA | 4.0 | 4.1 | 4.4 |
|  | M3 | MLA | 3.9 | 4.1 | 4.4 |
|  | M4 | MLA | 4.2 | 4.1 | 4.4 |
| RMCAH/CYA | M1 | MLA | 3.9 | 4.0 | 4.3 |
|  | M2 | MLA/CYA | 4.0 | 4.0 | 4.4 |
|  | M3 | MLA/CYA | 4.0 | 4.1 | 4.4 |
|  | M4 | MLA | 7.8 | 4.6 | 7.3 |
| RMCAH/ACE | M1 | MLA | 3.8 | 4.0 | 4.5 |
|  | M2 | ACE | 3.9 | 4.1 | 4.5 |
|  | M3 | ACE | 4.0 | 4.2 | 4.5 |
|  | M4 | ACE | 4.1 | 3.9 | 4.5 |
| RMCAH/BARa | M1 | MLA | 4.0 | 4.1 | 4.4 |
|  | M2* | BAR | 3.6 | 3.9 | 4.1 |
|  | M3* | BAR | 3.4 | 3.8 | 4.1 |
|  | M4* | BAR | 3.3 | 3.6 | 4.1 |
| RMCAH/BARb | M1 | MLA | 4.0 | 4.5 | 4.2 |
|  | M2 | MLA | 3.9 | 4.2 | 3.7 |
|  | M3* | BAR | 3.4 | 4.3 | 3.8 |
|  | M4* | BAR | 3.4 | 4.1 | 3.6 |

* In the monomers complexed with barbituric acid, there is no atom from the ligand in the active center of RMCAH close to the three serine γO atoms. The distances between the γO atoms are relatively smaller than those in RMCAH/MLA and RMCAH/ACE structures/monomers, whereas the position D is occupied.
