# Supplementary material for: Crystal structures of Moorella thermoacetica cyanuric acid hydrolase reveal conformational flexibility and asymmetry important for catalysis
Source: PLoS One. 2019 Jun 10;14(6):e0216979. doi: 10.1371/journal.pone.0216979 (PMC6557486; doi:10.1371/journal.pone.0216979)
Supplement: S4 Table — (DOCX) [file pone.0216979.s004.docx]

**S4 Table. Calcium coordination in the present structures and magnesium coordination in the AtzD structure reported earlier**

|  | RMCAH | | *AtzD |  |
| --- | --- | --- | --- | --- |
|  | Dist. to Ca^2+^ (Å) | Dist. to “Ca^2+^”** (Å) | Dist. to Mg^2+^ (Å) |  |
|  | (M1 of MLA form) | (M4 of “APO” form) |  |  |
| E296-OE2 | 2.39 | 2.59 | E297-OE2 | 2.56 |
| A345-O | 2.44 | 2.88 | A346-O | 2.42 |
| Q348-O | 2.40 | 2.70 | Q349-O | 2.32 |
| P350-O | 2.35 | 2.69 | P351-O | 2.42 |
|  |  |  |  |  |
| G353-O | 2.67 | 2.88 | G354-O | 2.42 |
| W445 | 2.72 | 3.09 | W2150 | 2.43 |

* AtzD structure is by Peat *et al*., 2013.

** The Ca^2+^ site can be modelled as being partially occupied by “Ca^2+^”.
