# Supplementary material for: Crystal structures of Moorella thermoacetica cyanuric acid hydrolase reveal conformational flexibility and asymmetry important for catalysis
Source: PLoS One. 2019 Jun 10;14(6):e0216979. doi: 10.1371/journal.pone.0216979 (PMC6557486; doi:10.1371/journal.pone.0216979)
Supplement: S1 Fig — (PDF) [file pone.0216979.s005.pdf]

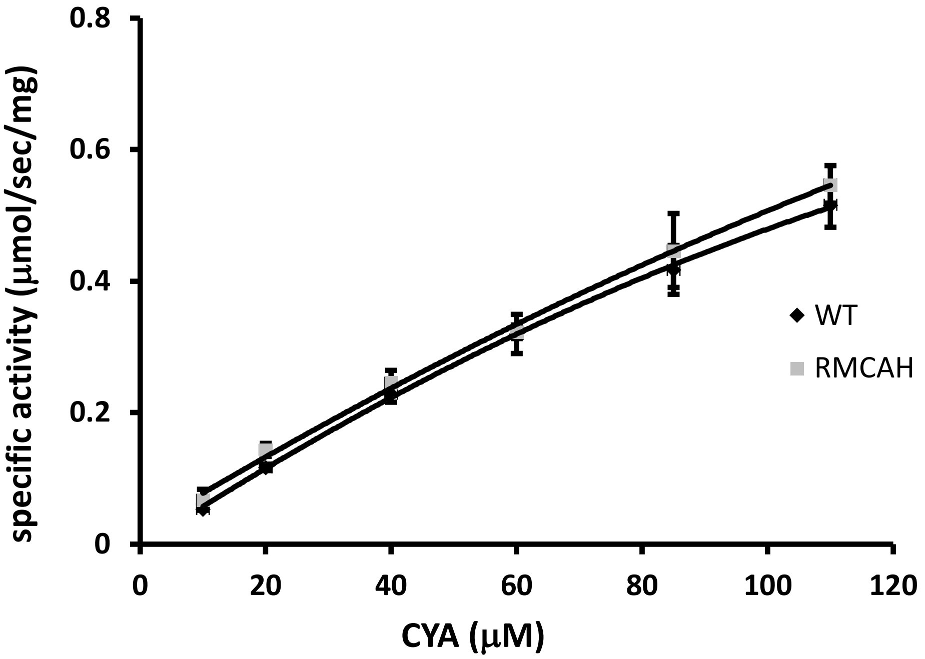

Kinetic constants for WT CAH and RMCAH.

	kcat	Km	kcat/Km
WT CAH	78 +/- 18 sec^{-1}	318 +/-95 μM	2.5 x 10^5 s^{-1},M^{-1}
RMCAH	73 +/- 17 sec^{-1}	276 +/- 86 μM	2.6 x 10^5 s^{-1},M^{-1}

S1 Fig. Comparison of WT CAH and RMCAH activities.
