# Supplementary material for: Crystal structures of Moorella thermoacetica cyanuric acid hydrolase reveal conformational flexibility and asymmetry important for catalysis
Source: PLoS One. 2019 Jun 10;14(6):e0216979. doi: 10.1371/journal.pone.0216979 (PMC6557486; doi:10.1371/journal.pone.0216979)
Supplement: S2 Fig — (PDF) [file pone.0216979.s006.pdf]

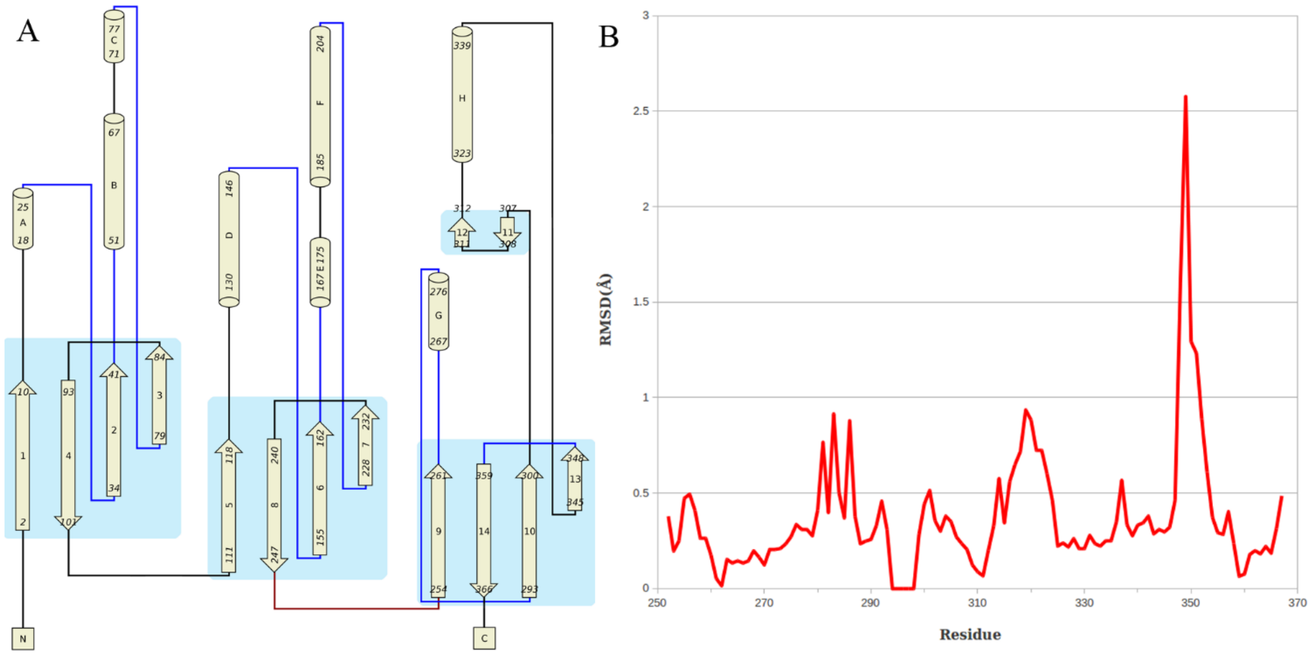


**S2 Fig. A**. 2D diagram of the 3-domain architecture of RMCAH, generated using Pro-origami (Stivala et al., 2011). Each domain consists a 4-stranded β-sheet. Domain 1 and 2 have three α-helices, whereas in domain 3, the helix 2 in domain 1 is changed to a small 2-pleated β-sheet. **B**. Per residue Cα distances between the open and closed conformation for domain 3. The segment with biggest r.m.s.d. connects the active site and the $Ca^{2+}$ binding site.

Stivala, A., Wybrow, M., Wirth, A., Whisstock, J. C., and Stuckey, P. J. (2011) Automatic generation of protein structure cartoons with Pro-origami. *Bioinformatics (Oxford, England)* **27**, 3315-3316
