# Supplementary material for: Crystal structures of Moorella thermoacetica cyanuric acid hydrolase reveal conformational flexibility and asymmetry important for catalysis
Source: PLoS One. 2019 Jun 10;14(6):e0216979. doi: 10.1371/journal.pone.0216979 (PMC6557486; doi:10.1371/journal.pone.0216979)
Supplement: S3 Fig — (PDF) [file pone.0216979.s007.pdf]

A.

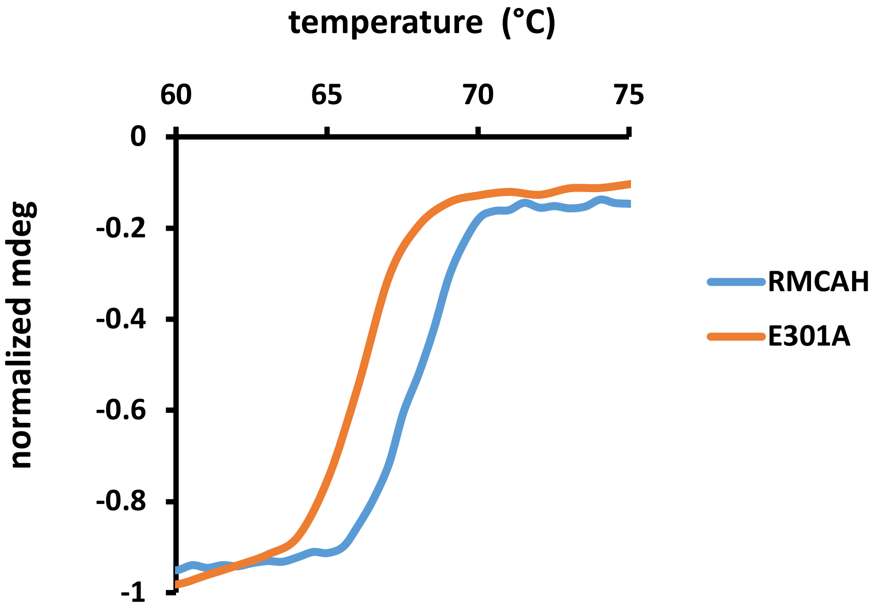

B.

	untreated	heat treated
**RMCAH**	**1.72 +/- .01**	**1.65 +/- 0.05 (96%)**
**E301A**	**1.56 +/- 0.06 (91%)**	**0.51 +/- 0.03 (30%)**

S3 Fig. Reduced stability of Ca^{2+} binding variant E301A. **A.** Thermal stability of RMCAH without (blue) or with E301A mutation (orange), determined by circular dichroism at 220 nm with 25 µM enzyme, 50 mM Tris pH 7.5, 200 mM NaCl. **B.** The effect of heat treatment (62 °C, 30 minutes) on the activity of E301A and WT RMCAH determined by the decrease in absorbance of cyanuric acid at 214 nm in 25mM Tris pH 7.0. Activity units are µmol CYA/min/mg CAH. Percent residual activity relative to that of untreated WT is given in parenthesis.
