# Supplementary material for: Crystal structures of Moorella thermoacetica cyanuric acid hydrolase reveal conformational flexibility and asymmetry important for catalysis
Source: PLoS One. 2019 Jun 10;14(6):e0216979. doi: 10.1371/journal.pone.0216979 (PMC6557486; doi:10.1371/journal.pone.0216979)
Supplement: S4 Fig — (PDF) [file pone.0216979.s008.pdf]

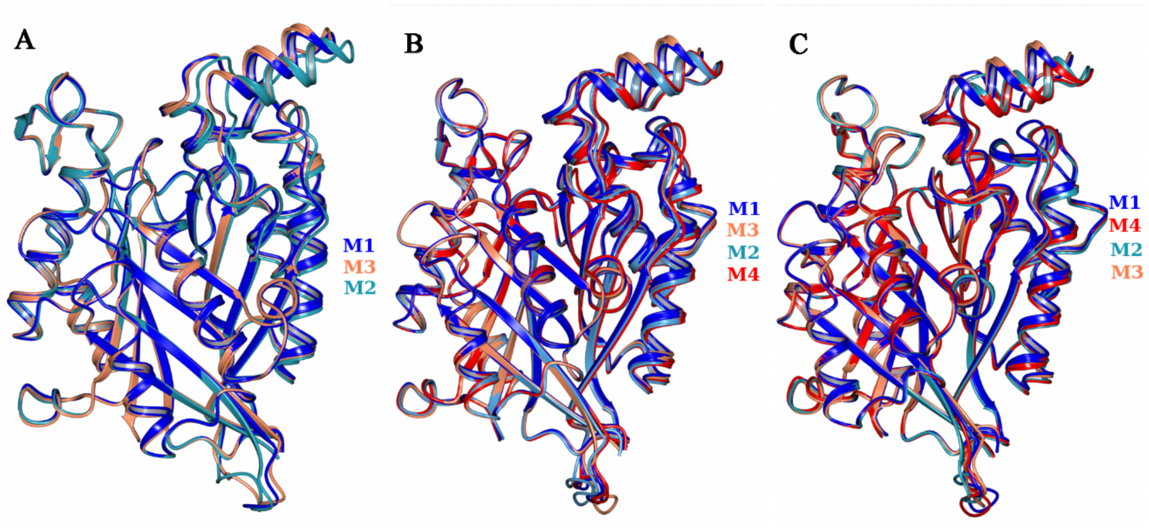

S4 Fig. Comparison of the structures of individual monomers for the RMCAH/CYA complex (A), RMCAH/ACE complex (B), and RMCAH/BAR complex (C). M4 in the RMCAH/CYA complex that takes the open (APO) conformation is omitted for clarity. Note variation between the monomers seen in the substrate entry channel, highlighted in Figures 5A and 6B.
