# Supplementary material for: Crystal structures of Moorella thermoacetica cyanuric acid hydrolase reveal conformational flexibility and asymmetry important for catalysis
Source: PLoS One. 2019 Jun 10;14(6):e0216979. doi: 10.1371/journal.pone.0216979 (PMC6557486; doi:10.1371/journal.pone.0216979)
Supplement: S5 Fig — (PDF) [file pone.0216979.s009.pdf]

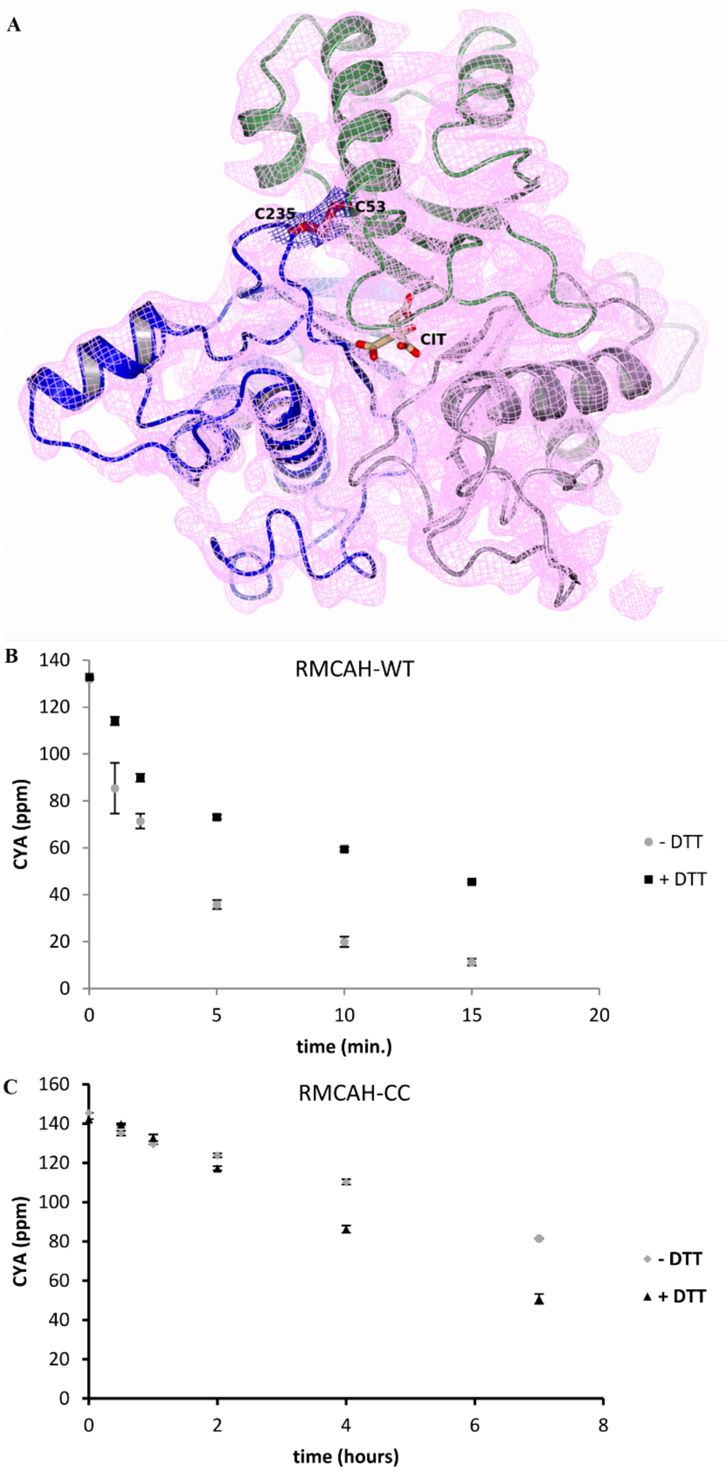

S5 Fig. A. The crystal structure of RMCAH (C46S/C162A/C218V/G53C/E235C) with the domain-2 locked in a partially open conformation, overlaid with the 2mFo-DFc electron density contoured at 1.0 σ. Blue mesh highlights electron density for the disulfide bond. A citrate molecule occupies the active site but electron density for the citrate is omitted for clarity. **B** and **C**. Activities of the re-engineered *Moorella* CAH (RMCAH) and its double mutant RMCAH-CC (G53C/E235C) tested in reducing (10 mM dithiothreitol) and non-reducing (no dithiothreitol) conditions.
